# Sample size and power analysis for ROC AUC differences in diagnostic tests: a methodological evaluation of the Obuchowski-McClish and Hanley-McNeil methods

**DOI:** 10.1186/s12874-026-02768-6

**Published:** 2026-01-28

**Authors:** Busra Emir, Fatma Ezgi Can, Elif Kaymaz, Zeynep Ozel, Mehmet Goktug Efgan, Mustafa Agah Tekindal, Ferhan Elmali

**Affiliations:** 1 Faculty of Medicine, Department of Biostatistics, Izmir Katip Celebi University, Izmir, 35620 Türkiye; 2 Institute of Health Sciences, Department of Biostatistics, Izmir Katip Celebi University, Izmir, Türkiye; 3 Faculty of Medicine, Department of Emergency Medicine, Izmir Katip Celebi University, Izmir, Türkiye

**Keywords:** Area under the curve, Diagnostic accuracy, Hanley-McNeil, Obuchowski-McClish, Power analysis, ROC curve, Sample size, Study design

## Abstract

**Background:**

Sample size determination for area under the curve (AUC) comparisons in diagnostic accuracy studies requires the consideration of multiple methodological parameters. The type of diagnostic test, the nature of the data (discrete or continuous), the correlation structure between tests, and the degree of AUC differences all influence optimal study design and planning. To address these factors, this study provides comprehensive sample size and power calculations for comparing AUCs between diagnostic tests across clinically relevant scenarios.

**Methods:**

We conducted a comprehensive evaluation of sample size and power analysis for AUC comparisons under varying correlation levels (ρ = 0.30, 0.50, 0.80), data types (discrete vs. continuous), and AUC differences (ΔAUC = 0.02–0.10). The Obuchowski–McClish method was applied for discrete data, and the Hanley–McNeil approach was applied for continuous data, assuming balanced case-control allocation and two-sided testing (*α* = 0.05). Sample sizes were calculated to achieve 80% statistical power based on established methodological approaches and adjusted for a 20% anticipated attrition rate. Power curves were generated to illustrate the relationship between sample size and statistical power across the evaluated scenarios.

**Results:**

The required sample sizes varied substantially across scenarios, ranging from 36 − 3,709 participants per group to achieve 80% power. The degree of difference in the area under the curve (AUC) was the primary determinant: ΔAUC = 0.02 required 909-3,709 participants, whereas a ΔAUC = 0.10 required only 36–142 participants. Inter-test correlation also had a marked impact on efficiency, with a strong correlation (ρ = 0.8) reducing sample sizes by 49% in discrete models and 68% in continuous models compared with a weak correlation (ρ = 0.3). Continuous data models consistently outperformed discrete models, requiring 24–53% fewer participants across all the scenarios. The most demanding scenario (ΔAUC = 0.02, discrete data, ρ = 0.3) required 3,709 participants per group, whereas the most efficient scenario (ΔAUC = 0.10, continuous data, ρ = 0.8) required only 36 participants, representing a 103-fold difference.

**Conclusions:**

Methodological choices lead to substantial variations in sample size requirements for diagnostic accuracy studies. Optimal parameter selection, particularly the use of continuous data models and accounting for strong inter-test correlations, can reduce the required sample sizes by up to 68% compared with suboptimal combinations. These results provide evidence-based guidance for efficient planning of diagnostic accuracy studies and underscore the critical importance of methodological considerations in study design optimization.

**Supplementary Information:**

The online version contains supplementary material available at 10.1186/s12874-026-02768-6.

## Background

The receiver operating characteristic (ROC) curve is the standard methodology for evaluating diagnostic test accuracy, with the area under the curve (AUC) serving as the principal summary measure of discriminatory performance [1, 2]. The AUC provides an intuitive interpretation of diagnostic ability, representing the probability that a randomly selected positive case will yield a higher test result than a randomly selected negative case [3]. Its use has become widespread across diagnostic research, ranging from biomarker discovery and validation to the development of clinical decision-making tools [4].

Current diagnostic research increasingly requires complex study designs. Emerging methodologies, including multireader multitest designs [5], metabolomic biomarker panels [6], and covariate-adjusted receiver operating characteristic (ROC) analysis [7], challenge traditional sample size calculation frameworks. Moreover, novel applications such as artificial intelligence-based diagnostic systems [8] and personalized medicine [9] have increased the demand for more sensitive and reliable methods for sample size determination.

Despite decades of methodological progress, determining appropriate sample sizes for AUC-based diagnostic studies remains a persistent challenge. Current approaches include nonparametric methods (e.g., DeLong et al.) [10], parametric frameworks (e.g., Obuchowski-McClish) [11], and scenario-specific techniques [12]. This methodological diversity offers flexibility but also introduces complexity, limiting practical applicability [13]. Jung (2024) has highlighted these limitations, noting that variance formulas often involve numerous asymptotically negligible terms and that specifying the many design parameters required for sample size calculations are practically infeasible [14].

Recent methodological advances, such as transformed ROC approaches [15], improved methods for cutoff point determination [16], Dunnett-type procedures for multiple comparisons [17] and bootstrap-based inference [18], further illustrate the evolving methodological landscape. Moreover, regulatory authorities and funders emphasize the need for more transparent justification of methodological choices. The updated Standards for Reporting of Diagnostic Accuracy Studies (STARD) statement [19] and the AI-specific STARD-AI protocol [20] underscore the need for careful methodological consideration in diagnostic accuracy studies.

This study systematically investigated the impact of various parameters on the sample size and power requirements for AUC comparisons. We evaluated correlation levels (ρ = 0.3, 0.5, 0.8), data types (discrete vs. continuous), and AUC differences (ΔAUC = 0.02–0.10) by applying the Obuchowski–McClish method for discrete data [21] and the Hanley–McNeil approach [22] for continuous data. Our results reveal up to 103-fold variation in required sample sizes depending on methodological choices, offering evidence-based guidance for researchers planning diagnostic test evaluation.

## Methods

### Study design

We conducted a comprehensive methodological study to evaluate the sample size required for AUC comparisons under varying methodological conditions. The study design incorporated factorial combinations of key parameters that influence sample size calculations in diagnostic accuracy research.

### Study parameters

We systematically evaluated parameters representing realistic clinical research scenarios. We assessed the required power and sample size for comparing AUCs across 30 distinct scenarios (5 AUC differences × 3 correlation levels × 2 data types).

Sample size group allocation ratio: Equal allocation was assumed for cases (n^+^) and controls (n^−^), representing optimal statistical efficiency for comparisons.

AUC values: Under the null hypothesis, we assumed a baseline diagnostic performance of AUC_1_ = 0.80, which is generally considered indicative of good discriminatory ability. For alternative scenarios, we evaluated AUC_2_ values ranging from 0.82 to 0.90, corresponding to clinically relevant improvements in diagnostic accuracy. These values reflect differences (ΔAUC = 0.02–0.10) spanning from minimal clinically important gains to substantial enhancements in diagnostic performance, as commonly reported in the medical literature [23, 24]. The analytical framework, however, is not limited to this value. The PASS-based procedure can be applied to alternative baseline AUC levels (e.g., 0.60–0.70) by specifying different AUC_1_-AUC_2_ combinations.

Correlation structures: In studies comparing two diagnostic tests on the same subjects, inter-test correlation refers to the extent to which the test results are related across individuals. When the same cases are evaluated by both tests, their outcomes are not independent. This correlation (denoted as ρ) affects the variance and the required sample size for comparing AUCs. The correlation coefficients (ρ = 0.3, 0.5, and 0.8) were chosen to represent weak, moderate, and strong inter-test relationships commonly observed in empirical diagnostic accuracy studies [1, 21]. The correlation level directly affects the variance of the AUC difference (ΔAUC); higher correlations reduce the variability and consequently the required sample size for a given power.

Data types: Both discrete and continuous data scenarios were examined to determine the diversity of diagnostic test formats encountered in clinical practice [23]. For discrete data scenarios, we applied the Obuchowski–McClish method [21], which accounts for the correlation structure between ROC curves and provides appropriate variance estimates for AUC differences in discrete diagnostic test comparisons. B_1_ and B_2_ were defined as the ratio of the standard deviation of the control group to that of the case group (SD_−_/SD_+_). For the analyses, the SDs were assumed to be equal to 1. The Hanley–McNeil approach [22] was used for continuous data scenarios. This method utilizes the asymptotic properties of AUC estimators and incorporates the correlation between measurements to determine the required sample sizes.

Lower and upper False Positive Rate (FPR): The lower and upper bounds of the probability that truly negative cases are incorrectly classified as positive (FPR) were 0 and 1, respectively.

Power calculations: All calculations were performed across statistical power levels ranging from 68% to 99.9% to accommodate diverse study requirements and resource constraints. These extended calculations were conducted to examine the actual power associated with a given sample size. In line with the conventional approach, studies are generally recommended to be initiated with a minimum of 80% statistical power. All calculations used two-sided hypothesis testing with a Type I error rate of α = 0.05, which is consistent with standard practice in diagnostic accuracy research.

### Output parameters

For each combination of parameters (ΔAUC, ρ, data types), we calculated the following:

Required sample size: The required sample size (n) per group assuming balanced case‒control allocation. This approach reflects common study designs in diagnostic accuracy research where equal numbers of case (n⁺) and control (n⁻) participants are enrolled.

Expected number of dropouts (ED): In our study, we assumed a dropout rate (DR) of 20%, reflecting the proportion of participants expected to be lost at random during the study, for whom no response data would be collected (i.e., treated as ‘missing’). Based on this assumption, the expected number of dropouts (ED) was defined analogously to D⁺, D⁻, and D.

Dropout inflated enrollment sample size (DISS): The adjusted number of participants to be enrolled ($\:{\mathrm{n}}^{{\prime\:}}$), accounting for anticipated participant attrition. Here, $\:{\mathrm{n}}^{+{\prime\:}}$ and $\:{\mathrm{n}}^{-{\prime\:}}$ denote the dropout-adjusted enrollment sizes for the case and control groups, respectively.

Target power (TP): The theoretical statistical power calculated from the analytical sample size formulas for a specified combination of parameters (ΔAUC, α, and ρ). It represents the expected probability of correctly rejecting the null hypothesis under ideal analytical assumptions.

Actual power (AP): The achieved empirical power, which accounts for practical constraints such as discrete sample sizes and the stochastic variability inherent in finite samples. To verify the accuracy of the analytical derivations, simulation-based validation was performed. 5,000 Monte Carlo replications were conducted for each scenario to estimate empirical power. In each iteration, paired diagnostic test results were simulated with predefined ΔAUC and correlation structures, and the rejection proportion of $\:{\mathrm{H}}_{0}:\:{\mathrm{A}\mathrm{U}\mathrm{C}}_{1}\:={\mathrm{A}\mathrm{U}\mathrm{C}}_{2}$ was recorded as AP.

The close agreement between TP and AP across different sample sizes and correlation levels confirmed the internal consistency and robustness of the analytical power calculations.

### Statistical analysis

All the methodological evaluations were conducted via PASS 2023 (power analysis and sample size software, version 23.0.1; NCSS LLC, Kaysville, Utah). PASS 2023 implements established analytical methods for AUC comparisons, utilizing the Obuchowski–McClish methodology for discrete data [21] and the Hanley–McNeil approach for continuous data [22]. Power curves were generated to visualize the relationship between sample size and statistical power, varying correlation structures and AUC differences.

### Statistical methodology

The primary aim of this study is to determine the required sample size and statistical power for comparing two correlated ROC curves. The variance of the difference between the two AUCs was estimated using methods appropriate to the measurement scale of the diagnostic test results. To ensure conceptual completeness, the theoretical framework of ROC analysis is briefly summarized, followed by detailed descriptions of the variance estimation procedures applied to discrete (rating) and continuous data. It should also be noted that this study is a methodological evaluation based on theoretical input parameters such as AUC₁, AUC₂, their difference, the inter-test correlation level, type of data (discrete, continuous) and the case-control ratio. No empirical data were analyzed. The binormal model appears as an underlying parametric framework required by the PASS algorithms for variance and sample size calculations, and not as a model fitted to real diagnostic measurements. Therefore, testing the binormal model assumption using statistical normality tests is not applicable within the design of this study.

Although Obuchowski and McClish (1997) originally developed variance and sample size formulas under the binormal model, which is typically associated with continuous test outcomes, the PASS software implements their rating-based variance formulas when diagnostic test results fall into discrete ordinal categories. The Hanley–McNeil (1983) [22] method is theoretically rooted in the Dorfman and Alf (1969) [25] framework for the empirical (nonparametric) AUC, rather than in the binormal model. PASS applies this method to continuous test outcomes and restricts its use to the full range of false-positive rates (FPRs). In this study, all variance estimation procedures were carried out using the methods provided in the “Tests for Two ROC Curves” procedure in PASS 2023 [26].

### Theoretical framework

This section summarizes the theoretical basis for comparing two correlated receiver operating characteristic (ROC) curves. Each area under the curve (AUC) represents the probability that a randomly selected case has a higher test result than a control. When two diagnostic tests are applied to the same subjects, their AUCs are statistically dependent because the same cases contribute to both estimates. This dependence is quantified as the inter-test correlation (ρ), which influences the variance of the AUC difference and statistical power and sample size requirements.

To compare two correlated AUCs, we tested the null hypothesis $\:{\mathrm{H}}_{0}:\:{\mathrm{A}\mathrm{U}\mathrm{C}}_{1}\:={\mathrm{A}\mathrm{U}\mathrm{C}}_{2}$ against the alternative hypothesis $\:{\mathrm{H}}_{1}:\:{\mathrm{A}\mathrm{U}\mathrm{C}}_{1}\:\ne\:{\mathrm{A}\mathrm{U}\mathrm{C}}_{2}$ using a two-sided Z test with a type I error rate (α) of 0.05. The test statistics and general formula for the required number of positive cases (N₊) are given by Eqs. (1) and (2).

1 $$\:\mathrm{Z}\:=\:\widehat{\Delta}/\sqrt{\mathrm{V}\mathrm{a}\mathrm{r}_0\left(\widehat{\Delta}\right)}$$

2 $$N_{+}\left(z_{\alpha}\sqrt{V_{0}\left(\hat{\Delta}\right)}+z_{\beta}\sqrt{V_{\mathrm{Alt}}\left(\hat{\Delta}\right)}\right)^{2}\bigg/\Delta^{2}$$

where $\widehat{\Delta}={\uptheta}_2-{\uptheta}_1$ represents the difference between the two AUCs and where $\:\mathrm{v}\mathrm{a}\mathrm{r}_0\left(\widehat{{\Delta\:}}\right)$ is the variance under the null hypothesis. Several approaches have been proposed for computing the AUC including the trapezoidal method and binormal model which computes the area by fitting two normal distributions to the data.

### Binormal model

The binormal model provides a mathematical framework for representing diagnostic test results when both the case and control groups follow approximately normal distributions with different means and variances. Under this assumption, each test’s performance is summarized by the probability that a randomly selected case has a higher test value than a randomly selected control. The model characterizes this probability using two key parameters A_i_ and B_i_ that capture the location and relative dispersion between the case and control distributions, respectively. These parameters allow analytical computation of the area under the ROC curve.

For continuous diagnostic test scores, where criterion variables followed a normal distribution $\:\mathrm{X}\:\sim\:\mathrm{N}\:({\upmu}\_,{\upsigma\:}\_^2)$ for the control group and $\:\mathrm{Y}\:\sim\:\mathrm{N}\:({\upmu\:}_{+},\:{\upsigma\:}_+^2)$ for the case group. The partial area under the curve was calculated via Eq. (3).

3 $$\:{{\uptheta\:}}_{\mathrm{i}}={\int\:}_{\mathrm{c}1}^{{\mathrm{c}}_{2}}{\Phi\:}({\mathrm{A}}_{\mathrm{i}}+{\mathrm{B}}_{\mathrm{i}}\mathrm{v}){\upvarphi\:}\left(\mathrm{v}\right)\mathrm{d}\mathrm{v}$$

where $\:{\Phi\:}\left(\mathrm{z}\right)$ denotes the cumulative standard normal distribution, $\:\mathrm{c}_\mathrm{j}\:=\:{\Phi}^{-1}\left(\mathrm{F}\mathrm{P}\mathrm{R}\mathrm{j}\right)$,

$\:\mathrm{A}\mathrm{ᵢ}\:=\:({{\upmu\:}}_{\mathrm{i}+}-{{\upmu\:}}_{\mathrm{i}-})/{\upsigma\:}_{\mathrm{i}+}$, and $\:\mathrm{B}\mathrm{ᵢ}\:=\:{{\upsigma\:}}_{\mathrm{i}-}/{{\upsigma\:}}_{\mathrm{i}+}$. For the full AUC, the integration was performed over the entire range of possible test values with the limits of integration set from $\:{\mathrm{c}}_{1}=\:-{\infty\:}$ to $\:{\mathrm{c}}_{2}={\infty\:}$. The binormal model presented to explain the theoretical basis of the ROC curve provides a parametric representation of the ROC curve. It is not directly used to estimate the variance of AUC differences.

### Variance of the AUC difference for discrete (Rating) data: Obuchowski–McClish methodology

When diagnostic test results are ordinal or rating-based (e.g., 1–5 or 1–7 scales), the variance of the difference between two correlated AUCs was computed using the method proposed by Obuchowski and McClish [21]. This approach provides analytic formulas for the variance and covariance of AUC estimates derived from discrete rating data and accounts for the paired nature of test results through correlation parameters for cases r₊ and r₋ that represent within subject dependencies between the two diagnostic tests in each subgroup. These parameters are incorporated into the covariance structure of the AUC difference to accurately reflect the shared variability between paired observations. Under the null hypothesis (H₀: AUC_1_ = AUC_2_) and the alternative hypothesis (H₁: AUC_1_ ≠ AUC_2_), the variances of the AUC difference $\:\left(\widehat{{\Delta\:}}\right)$ are computed using Eqs. (4) and (5).

4 $$\:{\mathrm{V}}_{0}\left(\widehat{{\Delta\:}}\right)=\mathrm{V}\left({\widehat{{\uptheta\:}}}_{1}\right)+\mathrm{V}\left({\widehat{{\uptheta\:}}}_{1}\right)-2\mathrm{C}({\widehat{{\uptheta\:}}}_{1},{\widehat{{\uptheta\:}}}_{1})$$

5 $$\:{\mathrm{V}}_{\mathrm{A}\mathrm{l}\mathrm{t}}\left(\widehat{{\Delta\:}}\right)=\mathrm{V}\left({\widehat{{\uptheta\:}}}_{1}\right)+\mathrm{V}\left({\widehat{{\uptheta\:}}}_{2}\right)-2\mathrm{C}({\widehat{{\uptheta\:}}}_{1},{\widehat{{\uptheta\:}}}_{2})$$

Equation (6) expresses the variance of each individual AUC estimate $\:\mathrm{V}\left({\widehat{{\uptheta\:}}}_{\mathrm{i}}\right)$ as a function of the location and scale parameters (A_i_, B_i_), and the ratio of control to case samples ($\:R={N}_{-}/{N}_{+}$).

6 $$\:\mathrm{V}\left({\widehat{{\uptheta\:}}}_{\mathrm{i}}\right)={\mathrm{f}}_{\mathrm{i}}^{2}\left(1+\frac{{\mathrm{B}}_{\mathrm{i}}^{2}}{\mathrm{R}}+\frac{{\mathrm{A}}_{\mathrm{i}}^{2}}{2}\right)+{\mathrm{g}}_{\mathrm{i}}^{2}\left({\mathrm{B}}_{\mathrm{i}}^{2}\left(\frac{1+\mathrm{R}}{2\mathrm{R}}\right)\right)$$

The covariance between the two correlated AUCs $\:\mathrm{C}\left({\widehat{{\uptheta\:}}}_{1},{\widehat{{\uptheta\:}}}_{2}\right)$ is given in Eq. (7), where $\:{\mathrm{r}}_{+}$ and $\:{\mathrm{r}}_{-}$ define the degree of correlation between the paired test results within the same cases and controls, respectively. This term quantifies how strongly the paired test outcomes move together within subjects.

7 $$\begin{aligned} \:\mathrm{C}\left({\widehat{{\uptheta\:}}}_{1},{\widehat{{\uptheta\:}}}_{2}\right)=&\:{\mathrm{f}}_{1}{\mathrm{f}}_{2}\left({\mathrm{r}}_{+}+{\mathrm{r}}_{-}\frac{{\mathrm{B}}_{1}{\mathrm{B}}_{2}}{\mathrm{R}}+{\mathrm{r}}_{+}^{2}\frac{{\mathrm{A}}_{1}{\mathrm{A}}_{2}}{2}\right)\\&+\frac{{\mathrm{g}}_{1}{\mathrm{g}}_{2}{\mathrm{B}}_{1}{\mathrm{B}}_{2}\left({\mathrm{r}}_{-}^{2} +\mathrm{R}{\mathrm{r}}_{+}^{2}\right)}{2\mathrm{R}}+\frac{{\mathrm{f}}_{1}{\mathrm{g}}_{2}{\mathrm{A}}_{1}{\mathrm{B}}_{2}{\mathrm{r}}_{+}^{2}}{2}\\&+\frac{{\mathrm{f}}_{2}{\mathrm{g}}_{1}{\mathrm{A}}_{2}{\mathrm{B}}_{1}{\mathrm{r}}_{+}^{2}}{2} \end{aligned}$$

Equations (8)-(13) define intermediate constants ($\:{\mathrm{f}}_{\mathrm{i}}$, $\:{\mathrm{g}}_{\mathrm{i}}$, $\:{\mathrm{E}}_{1\mathrm{i}}$-$\:{\mathrm{E}}_{4\mathrm{i}}$) that arise from the binormal model integrals and determine the shape of the ROC curve between specified false-positive rate (FPR) thresholds $\:{\mathrm{c}}_{1}$ and $\:{\mathrm{c}}_{2}$.

8 $$\:{\mathrm{f}}_{\mathrm{i}}=\frac{{\mathrm{E}}_{1\mathrm{i}}{\mathrm{E}}_{3\mathrm{i}}}{\sqrt{2{\uppi\:}{\mathrm{E}}_{2\mathrm{i}}}}$$

9 $$\:{\mathrm{g}}_{\mathrm{i}}=\frac{{\mathrm{E}}_{1\mathrm{i}}{\mathrm{E}}_{4\mathrm{i}}}{\sqrt{2{\uppi\:}{\mathrm{E}}_{2\mathrm{i}}}}-\frac{{\mathrm{A}}_{\mathrm{i}}{\mathrm{B}}_{\mathrm{i}}{\mathrm{E}}_{1\mathrm{i}}{\mathrm{E}}_{3\mathrm{i}}}{\sqrt{2{\uppi\:}{\mathrm{E}}_{2\mathrm{i}}^{3}}}$$

10 $$\:{\mathrm{E}}_{1\mathrm{i}}=\mathrm{e}\mathrm{x}\mathrm{p}\left(-\frac{{\mathrm{A}}_{\mathrm{i}}^{2}}{2+2{\mathrm{B}}_{\mathrm{i}}^{2}}\right)$$

11 $$\:{\mathrm{E}}_{2\mathrm{i}}=1+{\mathrm{B}}_{\mathrm{i}}^{2}$$

12 $$\:{\mathrm{E}}_{3\mathrm{i}}=\:{\Phi\:}\left({\mathrm{c}}_{2}\right)-{\Phi\:}\left({\mathrm{c}}_{1}\right)$$

13 $$\:{\mathrm{E}}_{4\mathrm{i}}=\mathrm{e}\mathrm{x}\mathrm{p}\left(-\frac{{\mathrm{c}}_{1}^{2}}{2}\right)-\mathrm{e}\mathrm{x}\mathrm{p}\left(-\frac{{\mathrm{c}}_{2}^{2}}{2}\right)$$

Equations (14)-(16) define the key parameters used to describe the distributional properties of the ROC curve. Specifically, Eq. (14) expresses cⱼ as the standard normal deviate corresponding to a given false positive rate ($\:{\mathrm{F}\mathrm{P}\mathrm{R}}_{\mathrm{j}}$), adjusted for the mean and variance differences between the case and control distributions. This transformation links the observed FPR values to the underlying z-score thresholds on the ROC curve.

14 $$\:{\mathrm{c}}_{\mathrm{j}}=\frac{{{\Phi\:}}^{-1}\left({\mathrm{F}\mathrm{P}\mathrm{R}}_{\mathrm{j}}\right)+\frac{{\mathrm{A}}_{\mathrm{j}}{\mathrm{B}}_{\mathrm{j}}}{1+{\mathrm{B}}_{\mathrm{j}}^{2}}}{\sqrt{1+{\mathrm{B}}_{\mathrm{j}}^{2}}}$$

Equation (15) defines R as the ratio of control to case subjects ($\:R={N}_{-}/{N}_{+}$), which balances the contribution of each group to the overall variance estimates, particularly when sample sizes are unequal.

15 $$\:R={N}_{-}/{N}_{+}$$

Equation (16) defines $\:{\mathrm{A}}_{\mathrm{i}}$, the location parameter that quantifies the separation between the case and control distributions for test i. This parameter depends on both the variance ratio ($\:{\mathrm{B}}_{\mathrm{i}}$) and the z-scores associated with the true negative (TNR) and false positive rates (FPR). $\:{\mathrm{T}\mathrm{N}\mathrm{R}}_{\mathrm{i}}$ denotes the cumulative proportion of controls scoring at or above a given ordinal category, $\:{\mathrm{F}\mathrm{P}\mathrm{R}}_{\mathrm{i}}$ represents the corresponding cumulative proportion of cases. $\:{\mathrm{A}}_{\mathrm{i}}$, $\:{\mathrm{B}}_{\mathrm{i}}$ and $\:R$ determine the shape and position of the ROC curve, thereby influencing the computation of AUC and its variance under the Obuchowski-McClish methodology [21].

16 $$\:{\mathrm{A}}_{\mathrm{i}}={\mathrm{B}}_{\mathrm{i}}{{\Phi\:}}^{-1}\left({\mathrm{T}\mathrm{N}\mathrm{R}}_{\mathrm{i}}\right)-{{\Phi\:}}^{-1}\left({\mathrm{F}\mathrm{P}\mathrm{R}}_{\mathrm{i}}\right)$$

The PASS documentation for the “Test for Two ROC Curves” procedure recommends the Obuchowski-McClish methodology for ordinal data, and this guidance was followed in the present study [26].

### Variance of the AUC difference for continuous data: Hanley-McNeil methodology

When diagnostic test results are continuously distributed, the variance of the difference between the two correlated AUCs was estimated using the classical approach of Hanley and McNeil [22]. Their methodology interprets the AUC as Wilcoxon statistics and derives variance and covariance components within the distribution-free framework originally established by Dorfman and Alf (1969) [25] for correlated ROC data. Under the assumption of continuous measurements, this method yields closed-form approximations for both the variance of each AUC and the covariance between paired AUC estimates. Consistent with the recommendations of the PASS Procedure User Manual, the Hanley-McNeil method was applied to all analyses involving continuous diagnostic test scores [26].

The total variance of the AUC difference $\:\widehat{{\Delta\:}}$ is given by Eq. (17).

17 $$\:\mathrm{V}\left(\widehat{{\Delta\:}}\right)=\mathrm{V}\left({\widehat{{\uptheta\:}}}_{1}\right)+\mathrm{V}\left({\widehat{{\uptheta\:}}}_{2}\right)-2\mathrm{C}({\widehat{{\uptheta\:}}}_{1},{\widehat{{\uptheta\:}}}_{2})$$

where the first two terms represent the variances of the individual AUC estimates, and the third term accounts for their covariance due to correlation between paired measurements.

The variance of each AUC $\:\mathrm{V}\left({\widehat{{\uptheta\:}}}_{\mathrm{i}}\right)$ is estimated according to Eq. (18).

18 $$\:\mathrm{V}\left({\widehat{{\uptheta\:}}}_{\mathrm{i}}\right)=\frac{{{\uptheta\:}}_{\mathrm{i}}}{\mathrm{R}\left(2-{{\uptheta\:}}_{\mathrm{i}}\right)}+\frac{2{{\uptheta\:}}_{\mathrm{i}}^{2}}{1+{{\uptheta\:}}_{\mathrm{i}}}-{{\uptheta\:}}_{\mathrm{i}}^{2}\left(\frac{1+\mathrm{R}}{\mathrm{R}}\right)$$

where $\:R={N}_{-}/{N}_{+}$ denotes the control-to-case ratio. This formulation links the AUC value directly to its sampling variability under continuous data assumptions.

The covariance term $\:\mathrm{C}\left({\widehat{{\uptheta\:}}}_{1},{\widehat{{\uptheta\:}}}_{2}\right)$ in Eq. (19) reflects the correlation (r) between the paired AUC estimates.

19 $$\:\mathrm{C}\left({\widehat{{\uptheta\:}}}_{1},{\widehat{{\uptheta\:}}}_{2}\right)=2\mathrm{r}\sqrt{\mathrm{V}\left({{\uptheta\:}}_{1}\right)\mathrm{V}\left({{\uptheta\:}}_{2}\right)}$$

Here, r represents the inter-test correlation between the two ROC areas derived from the same subjects. The value of r is obtained from the reference table provided by Hanley and McNeil [22] based on empirical simulations for varying levels of test correlation and disease prevalence.

Equations (17)-(19) demonstrate that the variability of the AUC difference depends on both the precision of the individual AUC estimates and the strength of the correlation between paired test results.

## Results

The sample size required for AUC comparisons varied dramatically across the evaluated scenarios, with methodological choices creating substantial differences in study efficiency. A comprehensive evaluation across different correlation levels, data types, and effect sizes revealed distinct patterns that provide practical guidance for planning studies on diagnostic accuracy.

### Effect of AUC difference values (ΔAUC)

The difference in the AUC between tests emerged as the primary determinant of sample size. For 80% power, larger effect sizes substantially reduced sample size requirements across all scenarios. ΔAUC = 0.02 required sample sizes ranging from 909 to 3709 participants per group across all the scenarios (Tables 1, 2, 3, 4, 5 and 6). For ΔAUC = 0.04, 225 to 919 participants were needed per group (Tables 1, 2, 3, 4, 5 and 6), whereas larger differences showed even greater efficacy: ΔAUC = 0.06 required 100–404 participants per group, ΔAUC = 0.08 needed 56–225 participants per group, and ΔAUC = 0.10 required only 36–142 participants per group (Supplementary Tables S1-S6).

### Effect of correlation between tests

Inter-test correlation strongly influenced the sample size requirements across all the scenarios. For the ΔAUC = 0.02 at 80% power in the discrete data models, a weak correlation (ρ = 0.3) required 3709 participants per group (Table 1), a moderate correlation (ρ = 0.5) needed 3018 participants per group (Table 3), and a strong correlation (ρ = 0.8) required 1899 participants per group (Table 5), representing a 49% reduction. This correlation effect was consistent across all effect sizes. For ΔAUC = 0.06, discrete models showed requirements from 404 participants per group at ρ = 0.3 to 211 participants per group at ρ = 0.8 (Supplementary Tables S1, S3, S5). For ΔAUC = 0.10, the range was 142 to 76 participants per group across correlation levels (Supplementary Tables S1, S3, S5).

For continuous data models with a ΔAUC = 0.02 at 80% power, the correlation effect was more pronounced. The sample sizes decreased from 2807 participants per group at ρ = 0.3 (Table 2) to 2092 participants per group at ρ = 0.5 (Table 4) and 909 participants per group at ρ = 0.8 (Table 6), representing a 68% reduction from weak to strong correlation. For larger effect sizes, ΔAUC = 0.06 showed a reduction from 304 to 100 participants per group (Supplementary Tables S2, S4, S6), whereas ΔAUC = 0.10 demonstrated a reduction from 107 to 36 participants per group across correlation levels.

**Table 1:** Sample size and statistical power for AUC comparisons (AUC₁=0.80 vs. AUC₂=0.82/0.84): discrete data type, equal SD ratios (B1 = B2 = 1.0), lower-upper FPR 0–1, and positive and negative group correlations 0.3

ΔAUC = 0.02								ΔAUC = 0.04							
Sample Size		ED		DISS				Sample Size		ED		DISS			
n^+^/n^−^	n	D^+^/D^−^	D	n^+’^/n^−’^	n^’^	TP	AP	n^+^/n^−^	n	D^+^/D^−^	D	n^+’^/n^−’^	n^’^	TP	AP
2793	5586	699	1398	3492	6984	0.680	0.680	694	1388	174	348	868	1736	0.680	0.680
2910	5820	728	1456	3638	7276	0.698	0.698	723	1446	181	362	904	1808	0.698	0.698
3026	6052	757	1514	3783	7566	0.715	0.715	752	1504	188	376	940	1880	0.715	0.716
3141	6282	786	1572	3927	7854	0.731	0.731	780	1560	195	390	975	1950	0.731	0.731
3253	6506	814	1628	4067	8134	0.746	0.746	807	1614	202	404	1009	2018	0.746	0.746
3371	6742	843	1686	4214	8428	0.761	0.761	836	1672	209	418	1045	2090	0.761	0.761
3478	6956	870	1740	4348	8696	0.774	0.774	862	1724	216	432	1078	2156	0.774	0.774
3599	7198	900	1800	4499	8998	0.788	0.788	892	1784	223	446	1115	2230	0.788	0.788
**3709**	**7418**	**928**	**1856**	**4637**	**9274**	**0.800**	**0.800**	**919**	**1838**	**230**	**460**	**1149**	**2298**	**0.800**	**0.800**
3825	7650	957	1914	4782	9564	0.812	0.812	947	1894	237	474	1184	2368	0.812	0.812
3936	7872	984	1968	4920	9840	0.823	0.823	975	1950	244	488	1219	2438	0.823	0.823
4054	8108	1014	2028	5068	10,136	0.834	0.834	1003	2006	251	502	1254	2508	0.834	0.834
4286	8572	1072	2144	5358	10,716	0.854	0.854	1060	2120	265	530	1325	2650	0.854	0.854
4400	8800	1100	2200	5500	11,000	0.863	0.863	1088	2176	272	544	1360	2720	0.863	0.863
4507	9014	1127	2254	5634	11,268	0.871	0.871	1114	2228	279	558	1393	2786	0.871	0.871
4620	9240	1155	2310	5775	11,550	0.879	0.879	1142	2284	286	572	1428	2856	0.879	0.879
4740	9480	1185	2370	5925	11,850	0.887	0.887	1171	2342	293	586	1464	2928	0.887	0.887
4852	9704	1213	2426	6065	12,130	0.894	0.894	1199	2398	300	600	1499	2998	0.894	0.894
**4970**	**9940**	**1243**	**2486**	**6213**	**12,426**	**0.901**	**0.901**	**1228**	**2456**	**307**	**614**	**1535**	**3070**	**0.901**	**0.901**
5078	10,156	1270	2540	6348	12,696	0.907	0.907	1254	2508	314	628	1568	3136	0.907	0.907
5192	10,384	1298	2596	6490	12,980	0.913	0.913	1282	2564	321	642	1603	3206	0.913	0.913
5313	10,626	1329	2658	6642	13,284	0.919	0.919	1311	2622	328	656	1639	3278	0.919	0.919
5421	10,842	1356	2712	6777	13,554	0.924	0.924	1338	2676	335	670	1673	3346	0.924	0.924
5535	11,070	1384	2768	6919	13,838	0.929	0.929	1366	2732	342	684	1708	3416	0.929	0.929
5657	11,314	1415	2830	7072	14,144	0.934	0.934	1395	2790	349	698	1744	3488	0.934	0.934
5761	11,522	1441	2882	7202	14,404	0.938	0.938	1421	2842	356	712	1777	3554	0.938	0.938
5900	11,800	1475	2950	7375	14,750	0.943	0.943	1455	2910	364	728	1819	3638	0.943	0.943
5989	11,978	1498	2996	7487	14,974	0.946	0.946	1477	2954	370	740	1847	3694	0.946	0.946
**6115**	**12,230**	**1529**	**3058**	**7644**	**15,288**	**0.950**	**0.950**	**1507**	**3014**	**377**	**754**	**1884**	**3768**	**0.950**	**0.950**
6216	12,432	1554	3108	7770	15,540	0.953	0.953	1532	3064	383	766	1915	3830	0.953	0.953
6287	12,574	1572	3144	7859	15,718	0.955	0.955	1549	3098	388	776	1937	3874	0.955	0.955
6477	12,954	1620	3240	8097	16,194	0.960	0.960	1596	3192	399	798	1995	3990	0.960	0.960
8626	17,252	2157	4314	10,783	21,566	0.990	0.990	2120	4240	530	1060	2650	5300	0.990	0.990
**11,952**	**23,904**	**2988**	**5976**	**14,940**	**29,880**	**0.999**	**0.999**	**2931**	**5862**	**733**	**1466**	**3664**	**7328**	**0.999**	**0.999**

Values shown in bold indicate the required sample sizes corresponding to statistical power levels of 0.80, 0.90, 0.95, and 0.99 under the specified AUC and dropout conditions

*ED* Expected number of dropouts, *DISS* Dropout-inflated enrollment sample size, *TP* Target power, *AP* Actual power

**Table 2:** Sample size and statistical power for AUC comparisons (AUC₁=0.80 vs. AUC₂=0.82/0.84): continuous data type, lower-upper FPR 0–1, positive, and negative group correlations 0.3

ΔAUC = 0.02								ΔAUC = 0.04							
Sample Size		ED		DISS				Sample Size		ED		DISS			
n^+^/n^−^	n	D^+^/D^−^	D	n^+’^/n^−’^	n^’^	TP	AP	n^+^/n^−^	n	D^+^/D^−^	D	n^+’^/n^−’^	n^’^	TP	AP
2118	4236	530	1060	2648	5296	0.680	0.680	525	1050	132	264	657	1314	0.680	0.680
2206	4412	552	1104	2758	5516	0.698	0.698	547	1094	137	274	684	1368	0.698	0.699
2293	4586	574	1148	2867	5734	0.715	0.715	568	1136	142	284	710	1420	0.715	0.715
2379	4758	595	1190	2974	5948	0.731	0.731	589	1178	148	296	737	1474	0.731	0.731
2464	4928	616	1232	3080	6160	0.746	0.746	609	1218	153	306	762	1524	0.746	0.746
2552	5104	638	1276	3190	6380	0.761	0.761	631	1262	158	316	789	1578	0.761	0.761
2633	5266	659	1318	3292	6584	0.774	0.774	651	1302	163	326	814	1628	0.774	0.775
2725	5450	682	1364	3407	6814	0.788	0.788	673	1346	169	338	842	1684	0.788	0.788
**2807**	**5614**	**702**	**1404**	**3509**	**7018**	**0.800**	**0.800**	**693**	**1386**	**174**	**348**	**867**	**1734**	**0.800**	**0.800**
2894	5788	724	1448	3618	7236	0.812	0.812	714	1428	179	358	893	1786	0.812	0.812
2978	5956	745	1490	3723	7446	0.823	0.823	734	1468	184	368	918	1836	0.823	0.823
3066	6132	767	1534	3833	7666	0.834	0.834	756	1512	189	378	945	1890	0.834	0.834
3241	6482	811	1622	4052	8104	0.854	0.854	798	1596	200	400	998	1996	0.854	0.854
3327	6654	832	1664	4159	8318	0.863	0.863	819	1638	205	410	1024	2048	0.863	0.863
3408	6816	852	1704	4260	8520	0.871	0.871	838	1676	210	420	1048	2096	0.871	0.871
3493	6986	874	1748	4367	8734	0.879	0.879	859	1718	215	430	1074	2148	0.879	0.879
3583	7166	896	1792	4479	8958	0.887	0.887	881	1762	221	442	1102	2204	0.887	0.887
3667	7334	917	1834	4584	9168	0.894	0.894	901	1802	226	452	1127	2254	0.894	0.894
**3756**	**7512**	**939**	**1878**	**4695**	**9390**	**0.901**	**0.901**	**923**	**1846**	**231**	**462**	**1154**	**2308**	**0.901**	**0.901**
3837	7674	960	1920	4797	9594	0.907	0.907	942	1884	236	472	1178	2356	0.907	0.907
3922	7844	981	1962	4903	9806	0.913	0.913	963	1926	241	482	1204	2408	0.913	0.913
4014	8028	1004	2008	5018	10,036	0.919	0.919	985	1970	247	494	1232	2464	0.919	0.919
4095	8190	1024	2048	5119	10,238	0.924	0.924	1005	2010	252	504	1257	2514	0.924	0.924
4181	8362	1046	2092	5227	10,454	0.929	0.929	1026	2052	257	514	1283	2566	0.929	0.929
4272	8544	1068	2136	5340	10,680	0.934	0.934	1048	2096	262	524	1310	2620	0.934	0.934
4350	8700	1088	2176	5438	10,876	0.938	0.938	1067	2134	267	534	1334	2668	0.938	0.938
4455	8910	1114	2228	5569	11,138	0.943	0.943	1092	2184	273	546	1365	2730	0.943	0.943
4522	9044	1131	2262	5653	11,306	0.946	0.946	1108	2216	277	554	1385	2770	0.946	0.946
**4616**	**9232**	**1154**	**2308**	**5770**	**11,540**	**0.950**	**0.950**	**1131**	**2262**	**283**	**566**	**1414**	**2828**	**0.950**	**0.950**
4692	9384	1173	2346	5865	11,730	0.953	0.953	1149	2298	288	576	1437	2874	0.953	0.953
4745	9490	1187	2374	5932	11,864	0.955	0.955	1162	2324	291	582	1453	2906	0.955	0.955
4888	9776	1222	2444	6110	12,220	0.960	0.960	1197	2394	300	600	1497	2994	0.960	0.960
6503	13,006	1626	3252	8129	16,258	0.990	0.990	1587	3174	397	794	1984	3968	0.990	0.990
**9000**	**18,000**	**2250**	**4500**	**11,250**	**22,500**	**0.999**	**0.999**	**2189**	**4378**	**548**	**1096**	**2737**	**5474**	**0.999**	**0.999**

Values shown in bold indicate the required sample sizes corresponding to statistical power levels of 0.80, 0.90, 0.95, and 0.99 under the specified AUC and dropout conditions

*ED* Expected number of dropouts, *DISS* Dropout-inflated enrollment sample size, *TP* Target power, *AP* Actual power

### Data type comparisons

Compared with discrete data approaches, continuous data models consistently demonstrated superior efficiency across all correlation levels and effect sizes. For a ΔAUC = 0.02 at 80% power with a weak correlation (ρ = 0.3), continuous models required 2807 participants per group (Table 2) compared with 3709 participants for discrete models (Table 1), representing a 24% reduction in sample size.

The efficiency advantage of continuous models became more pronounced with stronger correlations. At ρ = 0.8 for ΔAUC = 0.02, continuous models required 909 participants per group (Table 6) versus 1899 participants for discrete models (Table 5), representing a 52% reduction in the required sample size.

This pattern was maintained across larger effect sizes. For a ΔAUC = 0.06, continuous models required 100 participants per group versus 211 participants for discrete models at ρ = 0.8 (Supplementary Tables S6 vs. S5), a 53% reduction. For ΔAUC = 0.08, the advantage was 56 versus 118 participants per group (52% reduction), and for ΔAUC = 0.10, continuous models needed 36 versus 76 participants per group (53% reduction) at strong correlation levels (Supplementary Tables S6 vs. S5).

**Table 3:** Sample size and statistical power for AUC comparisons (AUC₁=0.80 vs. AUC₂=0.82/0.84): discrete data type, equal SD ratios (B1 = B2 = 1.0), lower-upper FPR 0–1, and positive and negative group correlations 0.5

ΔAUC = 0.02								ΔAUC = 0.04							
Sample Size		ED		DISS				Sample Size		ED		DISS			
n^+^/n^−^	n	D^+^/D^−^	D	n^+’^/n^−’^	n^’^	TP	AP	n^+^/n^−^	n	D^+^/D^−^	D	n^+’^/n^−’^	n^’^	TP	AP
2271	4542	568	1136	2839	5678	0.680	0.680	565	1130	142	284	707	1414	0.680	0.680
2367	4734	592	1184	2959	5918	0.698	0.698	589	1178	148	296	737	1474	0.698	0.698
2461	4922	616	1232	3077	6154	0.715	0.715	612	1224	153	306	765	1530	0.715	0.715
2554	5108	639	1278	3193	6386	0.731	0.731	635	1270	159	318	794	1588	0.731	0.731
2646	5292	662	1324	3308	6616	0.746	0.746	658	1316	165	330	823	1646	0.746	0.746
2742	5484	686	1372	3428	6856	0.761	0.761	681	1362	171	342	852	1704	0.761	0.761
2829	5658	708	1416	3537	7074	0.774	0.774	703	1406	176	352	879	1758	0.774	0.774
2929	5858	733	1466	3662	7324	0.788	0.788	727	1454	182	364	909	1818	0.788	0.788
**3018**	**6036**	**755**	**1510**	**3773**	**7546**	**0.800**	**0.800**	**749**	**1498**	**188**	**376**	**937**	**1874**	**0.800**	**0.800**
3112	6224	778	1556	3890	7780	0.812	0.812	772	1544	193	386	965	1930	0.812	0.812
3203	6406	801	1602	4004	8008	0.823	0.823	795	1590	199	398	994	1988	0.823	0.823
3299	6598	825	1650	4124	8248	0.834	0.834	818	1636	205	410	1023	2046	0.834	0.834
3489	6978	873	1746	4362	8724	0.854	0.854	865	1730	217	434	1082	2164	0.854	0.854
3582	7164	896	1792	4478	8956	0.863	0.863	888	1776	222	444	1110	2220	0.863	0.863
3669	7338	918	1836	4587	9174	0.871	0.871	909	1818	228	456	1137	2274	0.871	0.871
3761	7522	941	1882	4702	9404	0.879	0.879	932	1864	233	466	1165	2330	0.879	0.879
3859	7718	965	1930	4824	9648	0.887	0.887	956	1912	239	478	1195	2390	0.887	0.887
3950	7900	988	1976	4938	9876	0.894	0.894	978	1956	245	490	1223	2446	0.894	0.894
**4046**	**8092**	**1012**	**2024**	**5058**	**10,116**	**0.901**	**0.901**	**1002**	**2004**	**251**	**502**	**1253**	**2506**	**0.901**	**0.901**
4134	8268	1034	2068	5168	10,336	0.907	0.907	1024	2048	256	512	1280	2560	0.907	0.907
4227	8454	1057	2114	5284	10,568	0.913	0.913	1047	2094	262	524	1309	2618	0.913	0.913
4326	8652	1082	2164	5408	10,816	0.919	0.919	1071	2142	268	536	1339	2678	0.919	0.919
4414	8828	1104	2208	5518	11,036	0.924	0.924	1092	2184	273	546	1365	2730	0.924	0.924
4507	9014	1127	2254	5634	11,268	0.929	0.929	1115	2230	279	558	1394	2788	0.929	0.929
4607	9214	1152	2304	5759	11,518	0.934	0.934	1140	2280	285	570	1425	2850	0.934	0.934
4692	9384	1173	2346	5865	11,730	0.938	0.938	1161	2322	291	582	1452	2904	0.938	0.938
4805	9610	1202	2404	6007	12,014	0.943	0.943	1189	2378	298	596	1487	2974	0.943	0.943
4878	9756	1220	2440	6098	12,196	0.946	0.946	1206	2412	302	604	1508	3016	0.946	0.946
**4980**	**9960**	**1245**	**2490**	**6225**	**12,450**	**0.950**	**0.950**	**1232**	**2464**	**308**	**616**	**1540**	**3080**	**0.950**	**0.950**
5063	10,126	1266	2532	6329	12,658	0.953	0.953	1252	2504	313	626	1565	3130	0.953	0.953
5120	10,240	1280	2560	6400	12,800	0.955	0.955	1266	2532	317	634	1583	3166	0.955	0.955
5275	10,550	1319	2638	6594	13,188	0.960	0.960	1304	2608	326	652	1630	3260	0.960	0.960
7029	14,058	1758	3516	8787	17,574	0.990	0.990	1734	3468	434	868	2168	4336	0.990	0.990
**9744**	**19,488**	**2436**	**4872**	**12,180**	**24,360**	**0.999**	**0.999**	**2400**	**4800**	**600**	**1200**	**3000**	**6000**	**0.999**	**0.999**

Values shown in bold indicate the required sample sizes corresponding to statistical power levels of 0.80, 0.90, 0.95, and 0.99 under the specified AUC and dropout conditions

*ED* Expected number of dropouts, *DISS* Dropout-inflated enrollment sample size, *TP* Target power, *AP* Actual power

**Table 4:** Sample size and statistical power for *AUC* comparisons (*AUC*₁=0.80 vs. *AUC₂=*0.82/0.84): continuous data type, lower-upper FPR 0–1, positive, and negative group correlations 0.5

ΔAUC = 0.02								ΔAUC = 0.04								
Sample Size		ED		DISS				Sample Size			ED		DISS			
n^+^/n^−^	n	D^+^/D^−^	D	n^+’^/n^−’^	n^’^	TP	AP	n^+^/n^−^		n	D^+^/D^−^	D	n^+’^/n^−’^	n^’^	TP	AP
1578	3156	395	790	1973	3946	0.680	0.680	392		784	98	196	490	980	0.680	0.681
1644	3288	411	822	2055	4110	0.698	0.698	408		816	102	204	510	1020	0.698	0.699
1709	3418	428	856	2137	4274	0.715	0.715	424		848	106	212	530	1060	0.715	0.716
1773	3546	444	888	2217	4434	0.731	0.731	439		878	110	220	549	1098	0.731	0.731
1836	3672	459	918	2295	4590	0.746	0.746	455		910	114	228	569	1138	0.746	0.747
1902	3804	476	952	2378	4756	0.761	0.761	471		942	118	236	589	1178	0.761	0.762
1962	3924	491	982	2453	4906	0.774	0.774	485		970	122	244	607	1214	0.774	0.774
2031	4062	508	1016	2539	5078	0.788	0.788	502		1004	126	252	628	1256	0.788	0.788
**2092**	**4184**	**523**	**1046**	**2615**	**5230**	**0.800**	**0.800**	**517**		**1034**	**130**	**260**	**647**	**1294**	**0.800**	**0.800**
2157	4314	540	1080	2697	5394	0.812	0.812	533		1066	134	268	667	1334	0.812	0.813
2219	4438	555	1110	2774	5548	0.823	0.823	548		1096	137	274	685	1370	0.823	0.823
2285	4570	572	1144	2857	5714	0.834	0.834	564		1128	141	282	705	1410	0.834	0.834
2416	4832	604	1208	3020	6040	0.854	0.854	596		1192	149	298	745	1490	0.854	0.854
2480	4960	620	1240	3100	6200	0.863	0.863	611		1222	153	306	764	1528	0.863	0.863
2540	5080	635	1270	3175	6350	0.871	0.871	626		1252	157	314	783	1566	0.871	0.871
2603	5206	651	1302	3254	6508	0.879	0.879	641		1282	161	322	802	1604	0.879	0.879
2671	5342	668	1336	3339	6678	0.887	0.887	658		1316	165	330	823	1646	0.887	0.887
2733	5466	684	1368	3417	6834	0.894	0.894	673		1346	169	338	842	1684	0.894	0.894
**2800**	**5600**	**700**	**1400**	**3500**	**7000**	**0.901**	**0.901**	**689**		**1378**	**173**	**346**	**862**	**1724**	**0.901**	**0.901**
2860	5720	715	1430	3575	7150	0.907	0.907	704		1408	176	352	880	1760	0.907	0.907
2924	5848	731	1462	3655	7310	0.913	0.913	719		1438	180	360	899	1798	0.913	0.913
2992	5984	748	1496	3740	7480	0.919	0.919	736		1472	184	368	920	1840	0.919	0.919
3052	6104	763	1526	3815	7630	0.924	0.924	750		1500	188	376	938	1876	0.924	0.924
3117	6234	780	1560	3897	7794	0.929	0.929	766		1532	192	384	958	1916	0.929	0.929
3185	6370	797	1594	3982	7964	0.934	0.934	782		1564	196	392	978	1956	0.934	0.934
3243	6486	811	1622	4054	8108	0.938	0.938	797		1594	200	400	997	1994	0.938	0.938
3321	6642	831	1662	4152	8304	0.943	0.943	816		1632	204	408	1020	2040	0.943	0.943
3371	6742	843	1686	4214	8428	0.946	0.946	828		1656	207	414	1035	2070	0.946	0.946
**3442**	**6884**	**861**	**1722**	**4303**	**8606**	**0.950**	**0.950**	**845**		**1690**	**212**	**424**	**1057**	**2114**	**0.950**	**0.950**
3498	6996	875	1750	4373	8746	0.953	0.953	858		1716	215	430	1073	2146	0.953	0.953
3538	7076	885	1770	4423	8846	0.955	0.955	868		1736	217	434	1085	2170	0.955	0.955
3644	7288	911	1822	4555	9110	0.960	0.960	894		1788	224	448	1118	2236	0.960	0.960
4849	9698	1213	2426	6062	12,124	0.990	0.990	1186		2372	297	594	1483	2966	0.990	0.990
**6712**	**13,424**	**1678**	**3356**	**8390**	**16,780**	**0.999**	**0.999**	**1636**		**3272**	**409**	**818**	**2045**	**4090**	**0.999**	**0.999**

Values shown in bold indicate the required sample sizes corresponding to statistical power levels of 0.80, 0.90, 0.95, and 0.99 under the specified AUC and dropout conditions

*ED* Expected number of dropouts, *DISS* Dropout-inflated enrollment sample size, *TP* Target power, *AP* Actual power.

### Combined effects of methodological parameters

The interaction between correlation strength, data type, and effect size created substantial variations in sample size requirements for 80% power. The most demanding scenario (ΔAUC = 0.02, discrete data, ρ = 0.3) required 3709 participants per group (Table 1), whereas the most efficient scenario across all evaluated parameters (ΔAUC = 0.10, continuous data, ρ = 0.8) needed only 36 participants per group (Supplementary Table S6). This represents a 103-fold difference in sample size requirements between suboptimal and optimal methodological choices.

For ΔAUC = 0.04, substantial variation persisted. ΔAUC = 0.04 at 80% power, and the sample sizes ranged from 919 participants per group (discrete data, ρ = 0.3; Table 1) to 225 participants per group (continuous data, ρ = 0.8; Table 6), maintaining a 4-fold difference across scenarios.

For ΔAUC = 0.06, the range was 404 − 100 participants per group (Supplementary Tables S1 vs. S6), whereas ΔAUC = 0.08 showed requirements from 225 − 56 participants per group across scenarios (Supplementary Tables S1 vs. S6).

These interactive effects demonstrate that optimal methodological choices can achieve dramatic efficiency gains. Even within the same effect size category, selecting continuous data models and accounting for strong inter-test correlations can reduce sample size requirements by 50–70% compared with suboptimal parameter combinations.

### Practical sample size ranges for 80% power

The results reveal substantial variation in practical sample size requirements for diagnostic accuracy studies, which achieve 80% power, with methodological choices creating dramatic differences across AUC difference values. For studies investigating small but clinically meaningful differences (ΔAUC = 0.02), discrete data models required 1899–3709 participants per group depending on correlation strength (Tables 1, 3 and 5), whereas continuous data models demonstrated superior efficacy, with 909–2807 participants per group (Tables 2, 4 and 6). Studies targeting moderate improvements (ΔAUC = 0.04) showed reduced requirements, with discrete models requiring 474–919 participants per group (Tables 1 and 3, and 5) and continuous models requiring 225–693 participants per group across correlation scenarios (Tables 2 and 4, and 6).

For larger AUC differences, sample size requirements decreased substantially while maintaining the advantages of continuous data approaches and strong inter-test correlations. For discrete models, studies detecting a ΔAUC of 0.06 required 211–404 participants per group (Supplementary Tables S1, S3, S5) and 100–304 participants per group for continuous models (Supplementary Tables S2, S4, S6).

Investigations targeting ΔAUC = 0.08 needed 118–225 participants per group for discrete approaches (Supplementary Tables S1, S3, S5) and 56–169 participants per group for continuous methods (Supplementary Tables S2, S4, S6), whereas studies examining large differences (ΔAUC = 0.10) required only 76–142 participants per group for discrete methods (Supplementary Tables S1, S3, S5) and 36–107 participants per group for continuous approaches (Supplementary Tables S2, S4, S6).

These findings demonstrate that researchers can optimize study efficiency through careful methodological planning, with a comprehensive range of 36-3709 participants per group, highlighting the critical importance of parameter selection in diagnostic accuracy study design. The consistent efficiency advantages of continuous data models and strong inter-test correlations provide clear guidance for maximizing statistical power while minimizing resource requirements.

### Dropout-adjusted sample sizes

Assuming a constant drop-out rate of 20%, the required enrollment sample sizes increased substantially across all the scenarios (Tables 1, 2, 3, 4, 5 and 6, Supplementary Tables S1-S6). The expected number of dropouts (ED) corresponded to approximately one-fifth of the target sample size, and the adjusted enrollment (DISS) values were inflated by approximately 25% to compensate for anticipated attrition. This adjustment was particularly critical for scenarios with small diagnostic performance differences (ΔAUC = 0.02), where sample size requirements were already high. In contrast, under larger differences (ΔAUC = 0.04), the relative impact of drop-out adjustment was less pronounced. Continuous data models consistently require fewer participants than discrete data models do, even after accounting for attrition, and higher correlation levels (ρ = 0.5–0.8) further reduce both ED and DISS scores. This highlights the importance of incorporating anticipated attrition into study planning to avoid underpowered designs.

### Power curves across AUC differences, correlation levels, and data types

**Fig. 1:**
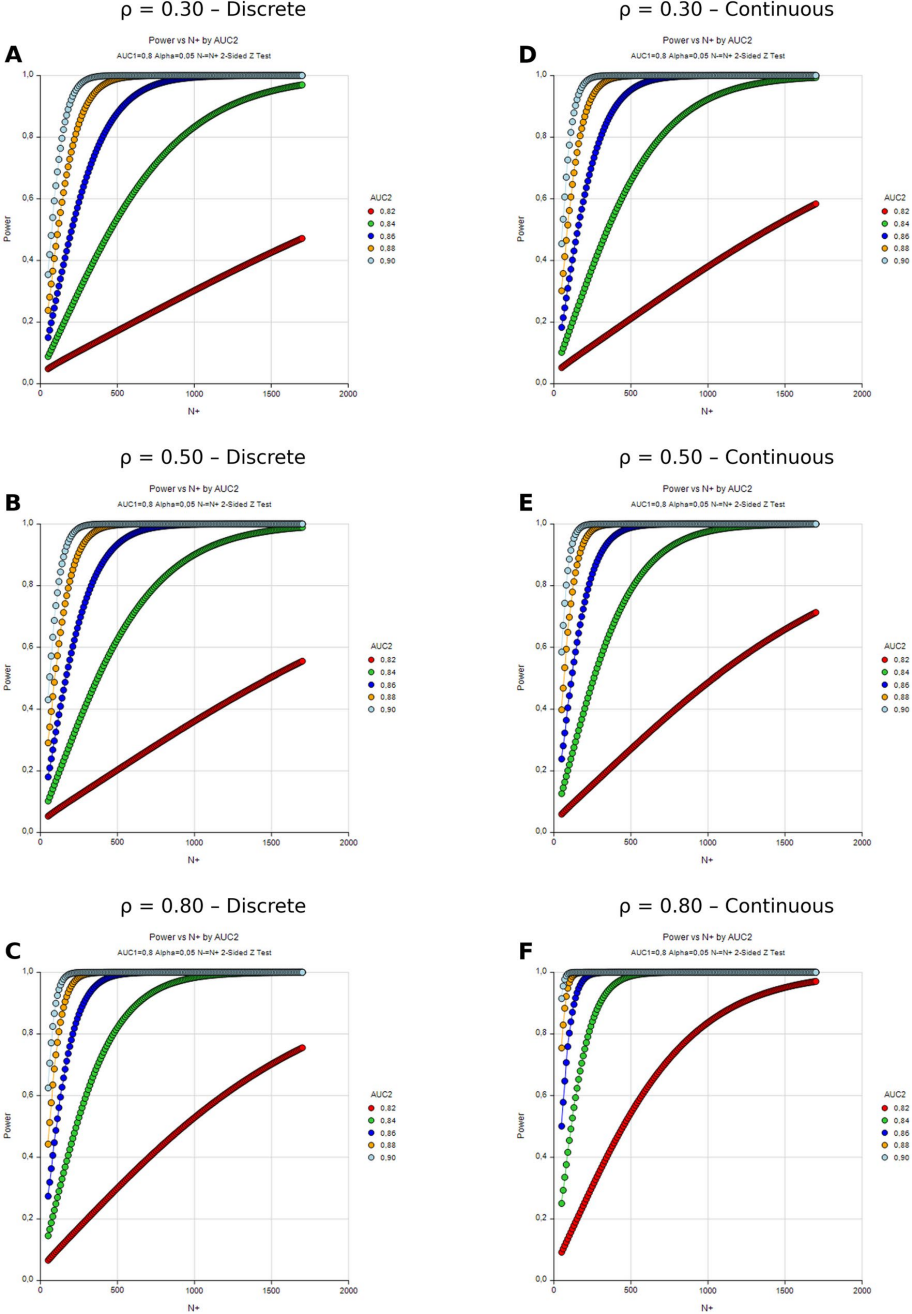
Power curves for sample size and statistical power across correlation levels and data types. Panels **A**, **B**, and **C** show results for discrete outcome data with correlation coefficients of ρ = 0.30, 0.50, and 0.80, respectively, whereas panels **D**, **E**, and **F** present the corresponding continuous outcome data. Each curve represents the power of detecting a difference in AUC between models assuming AUC_1_ = 0.80 and α = 0.05 (two tailed Z test)

Power curves demonstrate the relationship between sample size (N+) and statistical power for AUC_2_ values of 0.80, 0.82, 0.84, 0.86, 0.88, and 0.90 under different correlation scenarios. Panels A, B, and C represent discrete rating data with correlations of 0.30, 0.50, and 0.80, respectively; panels D, E, and F represent continuous data with corresponding correlation values. All analyses were conducted at a significance level of α = 0.05 with a two-tailed test (Fig. 1).

When all six panels are considered together, three main factors emerge as determinants of the relationship between sample size and statistical power: the magnitude of diagnostic performance improvement (AUC₂), the level of correlation (ρ), and the type of data (discrete vs. continuous).

For small improvements (e.g., AUC₂=0.82, ΔAUC = 0.02), the power curves increased slowly, with power remaining limited even at very large sample sizes. For example, under low correlation (ρ = 0.30; Fig. 1A), discrete data required approximately *n* = 9274 participants to reach 80% power (Table 1), whereas continuous data (Fig. 1D) achieved the same threshold at *n* ≈ 3509 (Table 2). In contrast, more pronounced improvements (AUC₂=0.86, 0.88, 0.90) produced steeper, classical S-shaped curves, reaching 80–95% power with considerably smaller sample sizes. For ΔAUC = 0.04, discrete data with ρ = 0.30 required *n* ≈ 2738 for 80% power (Table 1), whereas continuous data required only *n* ≈ 1498 (Table 2).

Under low correlation scenarios (ρ = 0.30; Fig. 1A and D), the power curves were shifted to the right, requiring the largest sample sizes among all settings. When correlation increased to ρ = 0.50 (Fig. 1B and E), the curves shifted leftward. For ΔAUC = 0.02, discrete data reached 80% power at *n* ≈ 7546 (Table 3), while continuous data required only *n* ≈ 2593 (Table 4). For ΔAUC = 0.04, 80% power required *n* ≈ 1984 for discrete data and *n* ≈ 1044 for continuous data (Tables 3 and 4).

Under high correlation (ρ = 0.80; Fig. 1C and F), power accumulation was markedly accelerated, allowing high power to be attained with relatively small sample sizes. For ΔAUC = 0.02, discrete data achieved 80% power at *n* ≈ 4748 (Table 5), whereas continuous data required only *n* = 2774 (Table 6). For ΔAUC = 0.04, discrete and continuous data achieved 80% power at *n* ≈ 1987 and *n* ≈ 1654, respectively.

Continuous data panels (Fig. 1D and F) consistently demonstrated superior performance compared with discrete data panels (Fig. 1A and C) at the same correlation levels, a pattern quantitatively reflected in the sample size reductions of 35–60% across Tables 1, 2, 3, 4, 5 and 6. In the continuous data models, the power curves were steeper, and target power levels were reached with substantially smaller sample sizes, with this efficiency advantage being most evident at the 80–90% power.

**Table 5:** Sample size and statistical power for AUC comparisons (AUC₁=0.80 vs. AUC₂=0.82/0.84): discrete data type, equal SD ratios (B1 = B2 = 1.0), lower-upper FPR 0–1, and positive and negative group correlations 0.8

ΔAUC = 0.02								ΔAUC = 0.04								
Sample Size		ED		DISS				Sample Size			ED		DISS			
n^+^/n^−^	n	D^+^/D^−^	D	n^+’^/n^−’^	n^’^	TP	AP	n^+^/n^−^		n	D^+^/D^−^	D	n^+’^/n^−’^	n^’^	TP	AP
1427	2854	357	714	1784	3568	0.680	0.680	357		714	90	180	447	894	0.680	0.681
1487	2974	372	744	1859	3718	0.698	0.698	372		744	93	186	465	930	0.698	0.699
1547	3094	387	774	1934	3868	0.715	0.715	387		774	97	194	484	968	0.715	0.716
1606	3212	402	804	2008	4016	0.731	0.731	401		802	101	202	502	1004	0.731	0.731
1664	3328	416	832	2080	4160	0.746	0.746	416		832	104	208	520	1040	0.746	0.747
1724	3448	431	862	2155	4310	0.761	0.761	431		862	108	216	539	1078	0.761	0.762
1780	3560	445	890	2225	4450	0.774	0.774	445		890	112	224	557	1114	0.774	0.775
1842	3684	461	922	2303	4606	0.788	0.788	460		920	115	230	575	1150	0.788	0.788
**1899**	**3798**	**475**	**950**	**2374**	**4748**	**0.800**	**0.800**	**474**		**948**	**119**	**238**	**593**	**1186**	**0.800**	**0.800**
1959	3918	490	980	2449	4898	0.812	0.812	489		978	123	246	612	1224	0.812	0.812
2016	4032	504	1008	2520	5040	0.823	0.823	503		1006	126	252	629	1258	0.823	0.823
2077	4154	520	1040	2597	5194	0.834	0.834	519		1038	130	260	649	1298	0.834	0.835
2197	4394	550	1100	2747	5494	0.854	0.854	548		1096	137	274	685	1370	0.854	0.854
2256	4512	564	1128	2820	5640	0.863	0.863	563		1126	141	282	704	1408	0.863	0.863
2311	4622	578	1156	2889	5778	0.871	0.871	577		1154	145	290	722	1444	0.871	0.871
2369	4738	593	1186	2962	5924	0.879	0.879	591		1182	148	296	739	1478	0.879	0.879
2431	4862	608	1216	3039	6078	0.887	0.887	607		1214	152	304	759	1518	0.887	0.887
2489	4978	623	1246	3112	6224	0.894	0.894	621		1242	156	312	777	1554	0.894	0.894
**2550**	**5100**	**638**	**1276**	**3188**	**6376**	**0.901**	**0.901**	**636**		**1272**	**159**	**318**	**795**	**1590**	**0.901**	**0.901**
2606	5212	652	1304	3258	6516	0.907	0.907	650		1300	163	326	813	1626	0.907	0.907
2665	5330	667	1334	3332	6664	0.913	0.913	665		1330	167	334	832	1664	0.913	0.913
2727	5454	682	1364	3409	6818	0.919	0.919	680		1360	170	340	850	1700	0.919	0.919
2783	5566	696	1392	3479	6958	0.924	0.924	694		1388	174	348	868	1736	0.924	0.924
2842	5684	711	1422	3553	7106	0.929	0.929	709		1418	178	356	887	1774	0.929	0.929
2905	5810	727	1454	3632	7264	0.934	0.934	725		1450	182	364	907	1814	0.934	0.934
2959	5918	740	1480	3699	7398	0.938	0.938	738		1476	185	370	923	1846	0.938	0.938
3031	6062	758	1516	3789	7578	0.943	0.943	756		1512	189	378	945	1890	0.943	0.943
3077	6154	770	1540	3847	7694	0.946	0.946	767		1534	192	384	959	1918	0.946	0.946
**3142**	**6284**	**786**	**1572**	**3928**	**7856**	**0.950**	**0.950**	**784**		**1568**	**196**	**392**	**980**	**1960**	**0.950**	**0.950**
3194	6388	799	1598	3993	7986	0.953	0.953	797		1594	200	400	997	1994	0.953	0.953
3230	6460	808	1616	4038	8076	0.955	0.955	806		1612	202	404	1008	2016	0.955	0.955
3329	6658	833	1666	4162	8324	0.960	0.960	830		1660	208	416	1038	2076	0.960	0.960
4440	8880	1110	2220	5550	11,100	0.990	0.990	1107		2214	277	554	1384	2768	0.990	0.990
**6162**	**12,324**	**1541**	**3082**	**7703**	**15,406**	**0.999**	**0.999**	**1535**		**3070**	**384**	**768**	**1919**	**3838**	**0.999**	**0.999**

Values shown in bold indicate the required sample sizes corresponding to statistical power levels of 0.80, 0.90, 0.95, and 0.99 under the specified AUC and dropout conditions

*ED* Expected number of dropouts, *DISS* Dropout-inflated enrollment sample size, *TP* Target power, *AP* Actual power

**Table 6:** Sample size and statistical power for AUC comparisons (AUC₁=0.80 vs. AUC₂=0.82/0.84): continuous data type, lower-upper FPR 0–1, positive, and negative group correlations 0.8

ΔAUC = 0.02								ΔAUC = 0.04							
Sample Size		ED		DISS				Sample Size		ED		DISS			
n^+^/n^−^	n	D^+^/D^−^	D	n^+’^/n^−’^	n^’^	TP	AP	n^+^/n^−^	n	D^+^/D^−^	D	n^+’^/n^−’^	n^’^	TP	AP
685	1370	172	344	857	1714	0.680	0.680	171	342	43	86	214	428	0.680	0.682
714	1428	179	358	893	1786	0.698	0.699	178	356	45	90	223	446	0.698	0.700
742	1484	186	372	928	1856	0.715	0.715	185	370	47	94	232	464	0.715	0.717
770	1540	193	386	963	1926	0.731	0.731	191	382	48	96	239	478	0.731	0.731
797	1594	200	400	997	1994	0.746	0.746	198	396	50	100	248	496	0.746	0.747
826	1652	207	414	1033	2066	0.761	0.761	205	410	52	104	257	514	0.761	0.762
852	1704	213	426	1065	2130	0.774	0.774	212	424	53	106	265	530	0.774	0.776
882	1764	221	442	1103	2206	0.788	0.788	219	438	55	110	274	548	0.788	0.789
**909**	**1818**	**228**	**456**	**1137**	**2274**	**0.800**	**0.800**	**225**	**450**	**57**	**114**	**282**	**564**	**0.800**	**0.800**
937	1874	235	470	1172	2344	0.812	0.812	232	464	58	116	290	580	0.812	0.812
964	1928	241	482	1205	2410	0.823	0.823	239	478	60	120	299	598	0.823	0.824
993	1986	249	498	1242	2484	0.834	0.834	246	492	62	124	308	616	0.834	0.835
1049	2098	263	526	1312	2624	0.854	0.854	260	520	65	130	325	650	0.854	0.855
1077	2154	270	540	1347	2694	0.863	0.863	267	534	67	134	334	668	0.863	0.864
1103	2206	276	552	1379	2758	0.871	0.871	273	546	69	138	342	684	0.871	0.871
1131	2262	283	566	1414	2828	0.879	0.879	280	560	70	140	350	700	0.879	0.880
1160	2320	290	580	1450	2900	0.887	0.887	287	574	72	144	359	718	0.887	0.887
1187	2374	297	594	1484	2968	0.894	0.894	294	588	74	148	368	736	0.894	0.895
**1216**	**2432**	**304**	**608**	**1520**	**3040**	**0.901**	**0.901**	**301**	**602**	**76**	**152**	**377**	**754**	**0.901**	**0.902**
1242	2484	311	622	1553	3106	0.907	0.907	307	614	77	154	384	768	0.907	0.907
1270	2540	318	636	1588	3176	0.913	0.913	314	628	79	158	393	786	0.913	0.913
1300	2600	325	650	1625	3250	0.919	0.919	321	642	81	162	402	804	0.919	0.919
1326	2652	332	664	1658	3316	0.924	0.924	328	656	82	164	410	820	0.924	0.925
1354	2708	339	678	1693	3386	0.929	0.929	335	670	84	168	419	838	0.929	0.930
1384	2768	346	692	1730	3460	0.934	0.934	342	684	86	172	428	856	0.934	0.934
1409	2818	353	706	1762	3524	0.938	0.938	348	696	87	174	435	870	0.938	0.938
1443	2886	361	722	1804	3608	0.943	0.943	356	712	89	178	445	890	0.943	0.943
1465	2930	367	734	1832	3664	0.946	0.946	362	724	91	182	453	906	0.946	0.946
**1496**	**2992**	**374**	**748**	**1870**	**3740**	**0.950**	**0.950**	**369**	**738**	**93**	**186**	**462**	**924**	**0.950**	**0.950**
1520	3040	380	760	1900	3800	0.953	0.953	375	750	94	188	469	938	0.953	0.953
1537	3074	385	770	1922	3844	0.955	0.955	379	758	95	190	474	948	0.955	0.955
1584	3168	396	792	1980	3960	0.960	0.960	391	782	98	196	489	978	0.960	0.960
2108	4216	527	1054	2635	5270	0.990	0.990	519	1038	130	260	649	1298	0.990	0.990
**2919**	**5838**	**730**	**1460**	**3649**	**7298**	**0.999**	**0.999**	**717**	**1434**	**180**	**360**	**897**	**1794**	**0.999**	**0.999**

Values shown in bold indicate the required sample sizes corresponding to statistical power levels of 0.80, 0.90, 0.95, and 0.99 under the specified AUC and dropout conditions

*ED* Expected number of Dropouts, *DISS* Drop-out-Inflated Enrollment Sample Size, *TP* Target Power, *AP*: Actual Power

## Discussion

This comprehensive evaluation demonstrated that methodological choices strongly influence sample size requirements and, consequently, the efficiency and feasibility of diagnostic accuracy studies. The observed 103-fold variation highlights the critical importance of careful parameter specification in study design. Our findings are in line with both the methodological literature and empirical clinical accuracy studies [27].

The degree of AUC difference (ΔAUC) emerged as the primary determinant of sample size. Small differences (ΔAUC = 0.02–0.05), which are common in the clinical literature, generally require large samples for single measurements and two-arm designs. AI-assisted reading in pulmonary embolism CT angiography improved the AUC by only 0.03 but required a carefully designed multireader multicase (MRMC) reader study with 18 readers and 240 cases to detect this effect [28, 29]. These findings are consistent with our estimates (225–919 participants per arm for ΔAUC = 0.04). In contrast, larger effects can be demonstrated with much smaller samples, as shown in the biomarker-based diagnosis of ovarian cancer, where a ΔAUC = 0.05–0.10 was detected with fewer than 100 participants [30]. Similar observations have been reported in neurodegenerative biomarker and traumatic brain injury studies, where marginal differences require multicenter cohorts ranging from several hundred to more than 1000 participants [31, 32].

Inter-test correlation structures (ρ) substantially reduce sample size requirements. A strong correlation (ρ = 0.8) led to nearly 50–70% reductions compared with a weak correlation (ρ = 0.3). These results emphasize the importance of pilot data or preliminary studies for estimating correlation structures accurately for reliable power calculations [33].

Continuous data consistently outperformed discrete ratings, yielding up to 50% reductions in the required sample size. This finding aligns with Obuchowski and McClish’s variance covariance formulations and with current reporting standards, which emphasize preregistration, explicit specification of the target ΔAUC, and transparent sample size planning [19, 20].

In exploratory biomarker studies where effect sizes are uncertain, researchers face a trade-off between comprehensiveness and feasibility. For studies comparing a new biomarker against an established assay with continuous measurements and expecting moderate correlation (ρ = 0.5), our results indicate that detecting ΔAUC = 0.06 requires 284 participants per group at 80% power (Supplementary Table S4). This sample size provides sufficient power for preliminary validation and is achievable in single-center studies. However, if only ordinal biomarker categories are available (e.g., low/medium/high expression levels), the required sample size increases to 413 participants per group, potentially requiring multicenter collaboration. Moore et al. (2010) validated the OVA1 multivariate biomarker panel for ovarian cancer using continuous risk scores from 516 patients and achieved sufficient power to detect improvements at ΔAUC = 0.05 compared to CA-125 alone [34]. In Alzheimer’s disease research, Palmqvist et al. (2020) showed that plasma phospho-tau217 outperformed other tau biomarkers using continuous concentration measurements in a cohort of 1,402 participants across multiple disease stages [35]. If these studies had relied on categorical biomarker classifications, significantly larger samples would have been required. These estimates could guide decisions about investing in quantitative assays or accepting the loss of efficiency of categorical measurements.

In rare diseases where case recruitment is severely limited, sample size optimization becomes critical for study feasibility. Consider the scenario of targeting ΔAUC = 0.08 between two diagnostic approaches with moderate correlation (ρ = 0.5). Continuous data models require only 158 cases (Supplementary Table 4), potentially achievable through a national registry collaboration, while discrete approaches require 230 cases (Supplementary Table 3), an increase of approximately 45% that would render the study unfeasible. In the diagnosis of amyotrophic lateral sclerosis (ALS), Poesen et al. (2017) compared neurofilament light chain measurements against traditional diagnostic criteria in a multicenter European cohort of 167 patients, using continuous protein concentration values to maximize statistical efficiency [36]. These findings suggest that investment in the development of continuous measurement protocols (e.g., quantitative imaging biomarkers or continuous laboratory assays) rather than categorical classifications for rare diseases may be essential to conduct adequately powered studies.

For screening test evaluation in large populations, where inclusion of each participant incurs direct costs, sample size optimization has significant economic implications. A screening trial targeting ΔAUC = 0.02 (representing a minimal but clinically meaningful improvement in population health) requires a minimum of 909–3,709 participants per group, depending on methodological choices (Tables 1, 2 and 5–6). This translates to total study costs ranging from €0.9 million to €7.4 million. The United Kingdom Ovarian Cancer Screening Collaboration Study (UKCTOCS) exemplifies this scenario, comparing multimodality screening strategies in 202,638 women with continuous CA-125 risk scores interpreted by the Ovarian Cancer Risk Algorithm (ROCA), requiring significant long-term follow-up costs [37]. The NELSON lung cancer screening trial enrolled 15,792 participants to identify small but significant improvements in CT-based nodule detection algorithms, and total costs exceeded €20 million over 10 years of follow-up [38]. Choosing continuous measures and designing studies to maximize inter-test correlation (e.g., within-subject comparisons rather than parallel groups) could reduce costs by €3–5 million. These resources could be redirected to implementation research or extended follow-up. These economic evaluations are particularly important for publicly funded research and health technology assessments.

Ethics committees for clinical research and other regulatory authorities require prospective justification of sample size based on predefined effect sizes and appropriate statistical methods. For diagnostic device approval studies, where ΔAUC = 0.05–0.08 is generally considered clinically meaningful, our results provide evidence-based sample size targets. A pivotal study comparing a new assay against a laboratory-based reference standard with an expected correlation of ρ = 0.5 and ΔAUC = 0.06 would require approximately 413 participants per group for discrete ordinal outcomes versus 284 participants for continuous measurements (Supplementary Table S3, Supplementary Table S4). The 45% increase for discrete data has implications not only for study duration and cost but also for regulatory timelines. Researchers should carefully consider whether measurement approaches can be designed to yield continuous rather than categorical outcomes to increase study efficiency while maintaining clinical validity.

The influence of underlying model assumptions should also be considered when interpreting our results. In the sample-size software PASS (Tests for Two ROC Curves module), the binormal ROC model is incorporated for discrete (rating) data the variance formulas derived by Obuchowski and McClish (1997) are used, for continuous data the classical Hanley-McNeil formulas apply. Although we use a binormal underlying conceptual model, when dealing with discrete rating data we rely on the Obuchowski-McClish adaptation of the model’s variance structure. Deviations from these assumptions are common in practical diagnostic research. Unbalanced or rare disease settings can lead to asymmetric variance contributions, leading to biased estimates of accuracy and required sample size estimates. Deviations from normality can affect the validity of binormal variance approximations, such as skewed or heavy-tailed test score distributions, and can reduce statistical power. Future research could assess the robustness of these results under relaxed assumptions through simulation studies or alternative distributions.

We recommend that future studies evaluating AUC comparisons justify their assumptions regarding the ΔAUC, correlation level ρ, and data type (discrete vs. continuous) and provide sample size calculations that are consistent with the methodological approach employed.

### Limitations

Our study has several limitations. We considered balanced case-control designs with equal sample sizes, which may not be generalizable to all diagnostic accuracy scenarios. In unbalanced designs such as rare disease settings where the number of positive cases is relatively small, the variance of the AUC estimate increases disproportionately, leading to potential underestimation of the required sample size when formulas derived under balanced assumptions are applied. Future studies could evaluate this bias empirically to quantify its direction and magnitude. We focused on two-sided testing, although certain diagnostic validation study designs may justify a one-sided approach depending on the clinical hypothesis. The correlation coefficients were restricted to 0.3, 0.5 and 0.8. Other dependence structures could be investigated to better represent test relationships in specific clinical data. Another limitation is that the actual power values computed in this study do not allow for the construction of 95% confidence intervals. Simulation based actual power was used to validate the behavior of the theoretical power curves, these simulations were intended for methodological verification rather than formal inference. Relaxing these assumptions and structures in future research would help assess the robustness of the approach and broaden its applicability to a wider range of diagnostic research.

## Conclusions

Study design parameters substantially influence sample size requirements for AUC comparisons in diagnostic accuracy research, with methodological choices leading to up to 103-fold differences. Continuous data models consistently provide efficiency advantages over discrete approaches, whereas strong inter-test correlations markedly reduce the required sample sizes.

These results offer practical, evidence-based guidance for researchers, emphasizing the need for methodological optimization to achieve both statistical precision and resource efficiency. By carefully specifying expected effect sizes, correlation structures, and analytical frameworks, investigators can design diagnostic accuracy studies that maintain adequate power while minimizing participant burden and resource demands. The observed variation in sample size underscores the critical importance of comprehensive methodological planning in the design of diagnostic accuracy research.

## Supplementary Information

Supplementary Material 1.

Supplementary Material 2.

Supplementary Material 3.

Supplementary Material 4.

Supplementary Material 5.

Supplementary Material 6.

Supplementary Material 7.

Supplementary Material 8.

## Data Availability

No dataset was generated or analyzed during the current study.

## Abbreviations

AP: Actual power
AUC: The area under the curve
ΔAUC: AUC differences
DISS: Drop-out inflated enrollment sample size
ED: Expected number of dropouts
FPR: False Positive Rate
ROC: Receiver operating characteristics
SD: Standard deviation
TP: Target power

## References

[1] Zhou XH, Obuchowski NA, McClish DK. Statistical methods in diagnostic medicine. 2nd ed. New York: Wiley; 2011.

[2] Hassanzad M, Hajian-Tilaki K. Methods of determining optimal cut-point of diagnostic biomarkers with application of clinical data in ROC analysis: an update review. BMC Med Res Methodol. 2024;24(1):84. 10.1186/s12874-024-02114-6. 38589814 10.1186/s12874-024-02198-2 PMC11000303

[3] Çorbacıoğlu ŞK, Aksel G. Receiver operating characteristic curve analysis in diagnostic accuracy studies: a guide to interpreting the area under the curve value. Turk J Emerg Med. 2023;23(4):195–8. 10.4103/tjem.tjem_182_23. 38024184 10.4103/tjem.tjem_182_23 PMC10664195

[4] Xia J, Broadhurst DI, Wilson M, Wishart DS. Translational biomarker discovery in clinical metabolomics: an introductory tutorial. Metabolomics. 2013;9(2):280–99. 10.1007/s11306-012-0482-9. 23543913 10.1007/s11306-012-0482-9 PMC3608878

[5] Kim E, Zhang Z, Wang Y, Zeng D. Power calculation for comparing diagnostic accuracies in a multireader, multitest design. Biometrics. 2014;70(4):1033–41. 10.1111/biom.12240. 25355470 10.1111/biom.12240 PMC4305439

[6] Liu D, Zhou XH. ROC analysis in biomarker combination with covariate adjustment. Acad Radiol. 2013;20(7):874–82. 10.1016/j.acra.2013.03.009. 23747153 10.1016/j.acra.2013.03.009 PMC3682803

[7] Inácio V, Rodríguez-Álvarez MX. The covariate-adjusted ROC curve: the concept and its importance, review of Inferential methods, and a new bayesian estimator. Stat Sci. 2022;37(4):541–61. 10.1214/21-STS839.

[8] Wei F, Huang P, Zhang B, Guo R, You X, Chen ZW, Chen Y. Machine learning analysis identified NNMT as a potential therapeutic target for hepatocellular carcinoma based on PCD-related genes. Sci Rep. 2025;15(1):7494. 10.1038/s41598-025-91625-5. 40032894 10.1038/s41598-025-91625-5 PMC11876361

[9] Okura K, Fukuyama K, Seo S, Nishino H, Yoh T, Shimoike N, et al. Personalized prognostic model for colorectal cancer in the era of precision medicine: a dynamic approach based on real-world data. Int J Clin Oncol. 2025;30(7):1376–85. 10.1007/s10147-025-02766-6. 40312604 10.1007/s10147-025-02766-6 PMC12187870

[10] Robin X, Turck N, Hainard A, Tiberti N, Lisacek F, Sanchez JC, Müller M. pROC: an open-source package for R and S + to analyze and compare ROC curves. BMC Bioinformatics. 2011;12:77. 10.1186/1471-2105-12-77. 21414208 10.1186/1471-2105-12-77 PMC3068975

[11] Obuchowski NA. Sample size calculations in studies of test accuracy. Stat Methods Med Res. 1998;7(4):371–92. 10.1177/096228029800700405. 9871953 10.1177/096228029800700405

[12] Hillis SL, Obuchowski NA, Berbaum KS. Power Estimation for multireader ROC methods: an updated and unified approach. Acad Radiol. 2011;18(2):129–42. 10.1016/j.acra.2010.10.007. 21232681 10.1016/j.acra.2010.09.007 PMC3053069

[13] Yang B, Olsen M, Vali Y, Langendam MW, Takwoingi Y, Hyde CJ, et al. Study designs for comparative diagnostic test accuracy: a methodological review and classification scheme. J Clin Epidemiol. 2021;138:128–38. 10.1016/j.jclinepi.2021.04.013. 33915262 10.1016/j.jclinepi.2021.04.013

[14] Jung SH. Sample size calculation for comparing two ROC curves. Pharm Stat. 2024;23(4):557–69. 10.1002/pst.2371. 38992978 10.1002/pst.2371

[15] Yang J, Kuan PF, Li X, Li J, Zhou XH. Transformed ROC curve for biomarker evaluation. Stat Med. 2024;43(30):5681–97. 10.1002/sim.10268. 39532385 10.1002/sim.10268

[16] Rota M, Antolini L, Valsecchi MG. Optimal cut-point definition in biomarkers: the case of censored failure time outcome. BMC Med Res Methodol. 2015;15:24. 10.1186/s12874-015-0009-y. 25887743 10.1186/s12874-015-0009-y PMC4430986

[17] Jung SH. A Dunnett-type test and its sample size calculation for comparing K ROC curves with a control. Diagnostics (Basel). 2024;14(16):1813. 10.3390/diagnostics14161813. 39202301 10.3390/diagnostics14161813 PMC11353566

[18] Blume JD. Bounding sample size projections for the area under a ROC curve. J Stat Plan Inference. 2009;139(3):711–21. 10.1016/j.jspi.2007.09.015. 20160839 10.1016/j.jspi.2007.09.015 PMC2631183

[19] Cohen JF, Korevaar DA, Altman DG, Bruns DE, Gatsonis CA, Hooft L, et al. STARD 2015 guidelines for reporting diagnostic accuracy studies: explanation and elaboration. BMJ Open. 2016;6(11):e012799. 10.1136/bmjopen-2016-012799. 28137831 10.1136/bmjopen-2016-012799 PMC5128957

[20] Sounderajah V, Ashrafian H, Golub RM, Shetty S, De Fauw J, Hooft L, et al. Developing a reporting guideline for artificial intelligence-centered diagnostic test accuracy studies: the STARD-AI protocol. BMJ Open. 2021;11(6):e047709. 10.1136/bmjopen-2020-047709. 34183345 10.1136/bmjopen-2020-047709 PMC8240576

[21] Obuchowski NA, McClish DK. Sample size determination for diagnostic accuracy studies involving binormal ROC curve indices. Stat Med. 1997;16(13):1529–42. 10.1002/(SICI)1097-0258(19970715)16:13%3C1529:AID-SIM566%3E3.0.CO;2-H. 9249923 10.1002/(sici)1097-0258(19970715)16:13<1529::aid-sim565>3.0.co;2-h

[22] Hanley JA, McNeil BJ. A method of comparing the areas under receiver operating characteristic curves derived from the same cases. Radiology. 1983;148(3):839–43. 10.1148/radiology.148.3.6878708. 6878708 10.1148/radiology.148.3.6878708

[23] Negida A, Fahim NK, Negida Y. Sample size calculation guide-part 4: how to calculate the sample size for a diagnostic test accuracy study based on sensitivity, specificity, and the area under the ROC curve. Adv J Emerg Med. 2019;3(3):e33. 10.22114/ajem.v0i0.158. 31410410 10.22114/ajem.v0i0.158 PMC6683590

[24] Søreide K, Kørner H, Søreide JA. Diagnostic accuracy and receiver-operating characteristics curve analysis in surgical research and decision making. Ann Surg. 2011;253(1):27–34. 10.1097/SLA.0b013e318204a892. 21294285 10.1097/sla.0b013e318204a892

[25] Dorfman DD, Alf E Jr. Maximum-likelihood Estimation of parameters of signal-detection theory and determination of confidence intervals rating-method data. J Math Psychol. 1969;6(3):487–96.

[26] NCSS LLC. (2024). *Tests for Two ROC Curves (PASS Procedure No. 265)* Kaysville, UT: NCSS, LLC. Retrieved from https://www.ncss.com/wp-content/themes/ncss/pdf/Procedures/PASS/Tests_for_Two_ROC_Curves.pdf

[27] DeLong ER, DeLong DM, Clarke-Pearson DL. Comparing the areas under two or more correlated receiver operating characteristic curves: a nonparametric approach. Biometrics. 1988;44(3):837–45. 10.2307/2531595. PMID:3203132. 3203132

[28] van Winkel SL, Rodríguez-Ruiz A, Appelman L, Teuwen J, Vreemann S, Karssemeijer N, et al. Impact of artificial intelligence support on accuracy and reading time in breast tomosynthesis image interpretation: a multireader multicase study. Eur Radiol. 2021;31(12):8682–91. 10.1007/s00330-021-07992-w. 33948701 10.1007/s00330-021-07992-w PMC8523448

[29] Obuchowski NA. Sample size tables for receiver operating characteristic studies. AJR Am J Roentgenol. 2000;175(3):603–8. 10.2214/ajr.175.3.1750603. 10954438 10.2214/ajr.175.3.1750603

[30] Wei SU, Li H, Zhang B. The diagnostic value of serum HE4 and CA-125 and ROMA index in ovarian cancer. Biomed Rep. 2016;5(1):41–4. 10.3892/br.2016.682. 27347403 10.3892/br.2016.682 PMC4906902

[31] Lantero-Rodriguez J, Vrillon A, Fernández-Lebrero A, Ortiz-Romero P, Snellman A, Montoliu-Gaya L, et al. Clinical performance and head-to-head comparison of CSF p-tau235 with p-tau181, p-tau217 and p-tau231 in two memory clinic cohorts. Alzheimers Res Ther. 2023;15(1):48. 10.1186/s13195-023-01201-0. 36899441 10.1186/s13195-023-01201-0 PMC9999575

[32] Papa L, McKinley WI, Valadka AB, Newman ZC, Nordgren RK, Pramuka PE, et al. Diagnostic performance of GFAP, UCH-L1, and MAP-2 within 30 and 60 minutes of traumatic brain injury. JAMA Netw Open. 2024;7(9):e2431115. 10.1001/jamanetworkopen.2024.31115. 39230905 10.1001/jamanetworkopen.2024.31115 PMC11375473

[33] Santini A, Man A, Voidazan S. Accuracy of diagnostic tests. J Crit Care Med (Targu Mures). 2021;7(3):241–8. 10.2478/jccm-2021-0022. 34722928 10.2478/jccm-2021-0022 PMC8519382

[34] Moore RG, McMeekin DS, Brown AK, DiSilvestro P, Miller MC, Allard WJ, et al. A novel multiple marker bioassay utilizing HE4 and CA125 for the prediction of ovarian cancer in patients with a pelvic mass. Gynecol Oncol. 2009;112(1):40–6. 10.1016/j.ygyno.2008.08.031. 18851871 10.1016/j.ygyno.2008.08.031 PMC3594094

[35] Palmqvist S, Janelidze S, Quiroz YT, Zetterberg H, Lopera F, Stomrud E, et al. Discriminative accuracy of plasma phospho-tau217 for alzheimer disease vs other neurodegenerative disorders. JAMA. 2020;324(8):772–81. 10.1001/jama.2020.12134. 32722745 10.1001/jama.2020.12134 PMC7388060

[36] Poesen K, De Schaepdryver M, Stubendorff B, Gille B, Muckova P, Wendler S, et al. Neurofilament markers for ALS correlate with extent of upper and lower motor neuron disease. Neurology. 2017;88(24):2302–9. 10.1212/WNL.0000000000004029. 28500227 10.1212/WNL.0000000000004029

[37] Jacobs IJ, Menon U, Ryan A, Gentry-Maharaj A, Burnell M, Kalsi JK, et al. Ovarian cancer screening and mortality in the UK collaborative trial of ovarian cancer screening (UKCTOCS): a randomised controlled trial. Lancet. 2016;387(10022):945–56. 10.1016/S0140-6736(15)01224-6. 26707054 10.1016/S0140-6736(15)01224-6 PMC4779792

[38] de Koning HJ, van der Aalst CM, de Jong PA, Scholten ET, Nackaerts K, Heuvelmans MA, et al. Reduced lung-cancer mortality with volume CT screening in a randomized trial. N Engl J Med. 2020;382(6):503–13. 10.1056/NEJMoa1911793. 31995683 10.1056/NEJMoa1911793

