# Supplementary figures and images for: Sample size and power analysis for ROC AUC differences in diagnostic tests: a methodological evaluation of the Obuchowski-McClish and Hanley-McNeil methods
Source: BMC Med Res Methodol. 2026 Jan 28;26:43. doi: 10.1186/s12874-026-02768-6 (PMC12924612; doi:10.1186/s12874-026-02768-6)

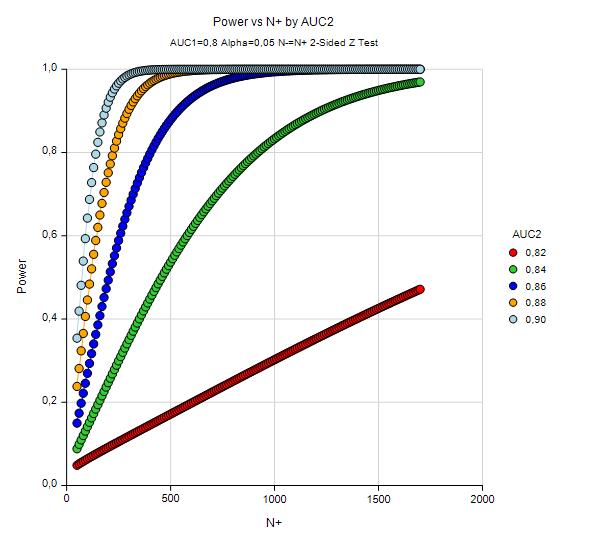

Supplement: Supplementary file 1 — Supplementary Material 1. [file 12874_2026_2768_MOESM1_ESM.jpeg]

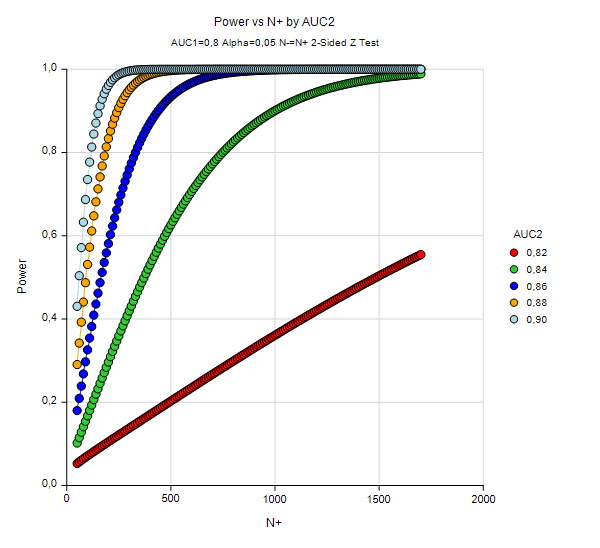

Supplement: Supplementary file 2 — Supplementary Material 2. [file 12874_2026_2768_MOESM2_ESM.jpeg]

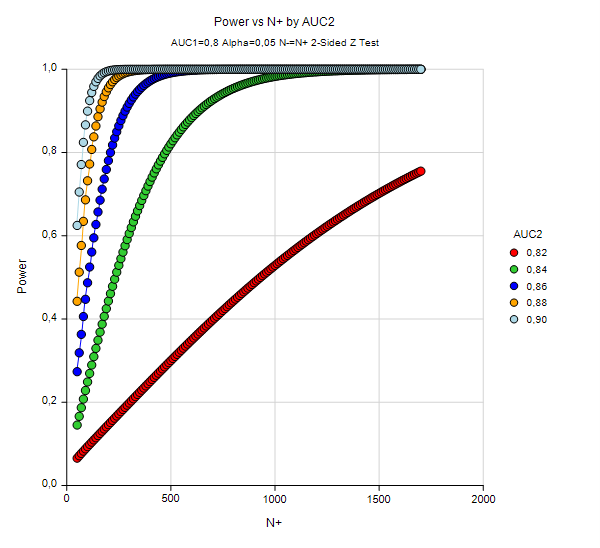

Supplement: Supplementary file 3 — Supplementary Material 3. [file 12874_2026_2768_MOESM3_ESM.jpeg]

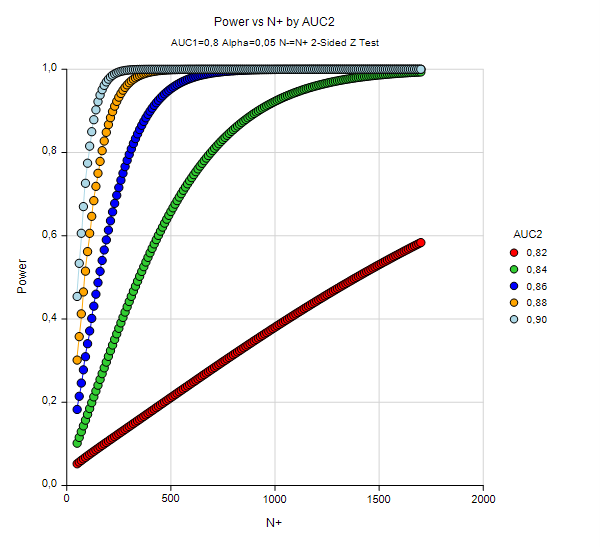

Supplement: Supplementary file 4 — Supplementary Material 4. [file 12874_2026_2768_MOESM4_ESM.jpeg]

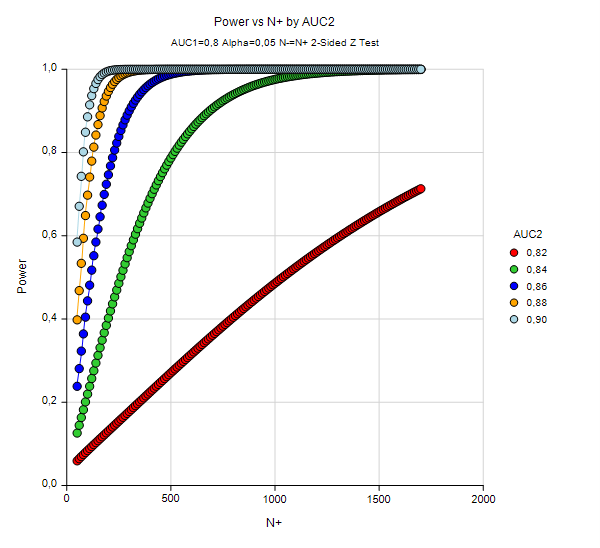

Supplement: Supplementary file 5 — Supplementary Material 5. [file 12874_2026_2768_MOESM5_ESM.jpeg]

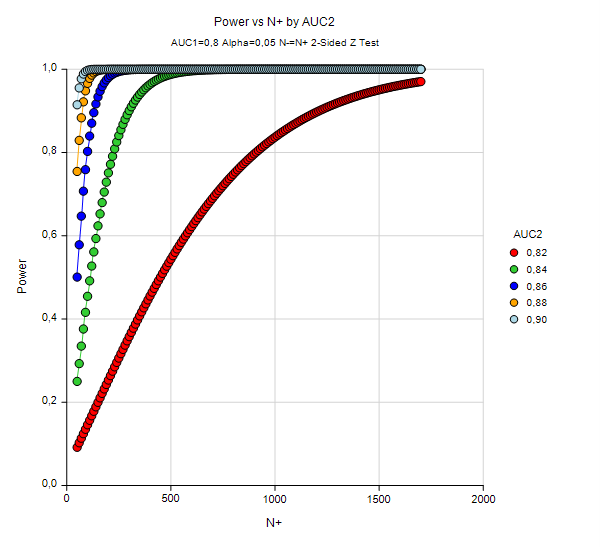

Supplement: Supplementary file 6 — Supplementary Material 6. [file 12874_2026_2768_MOESM6_ESM.jpeg]

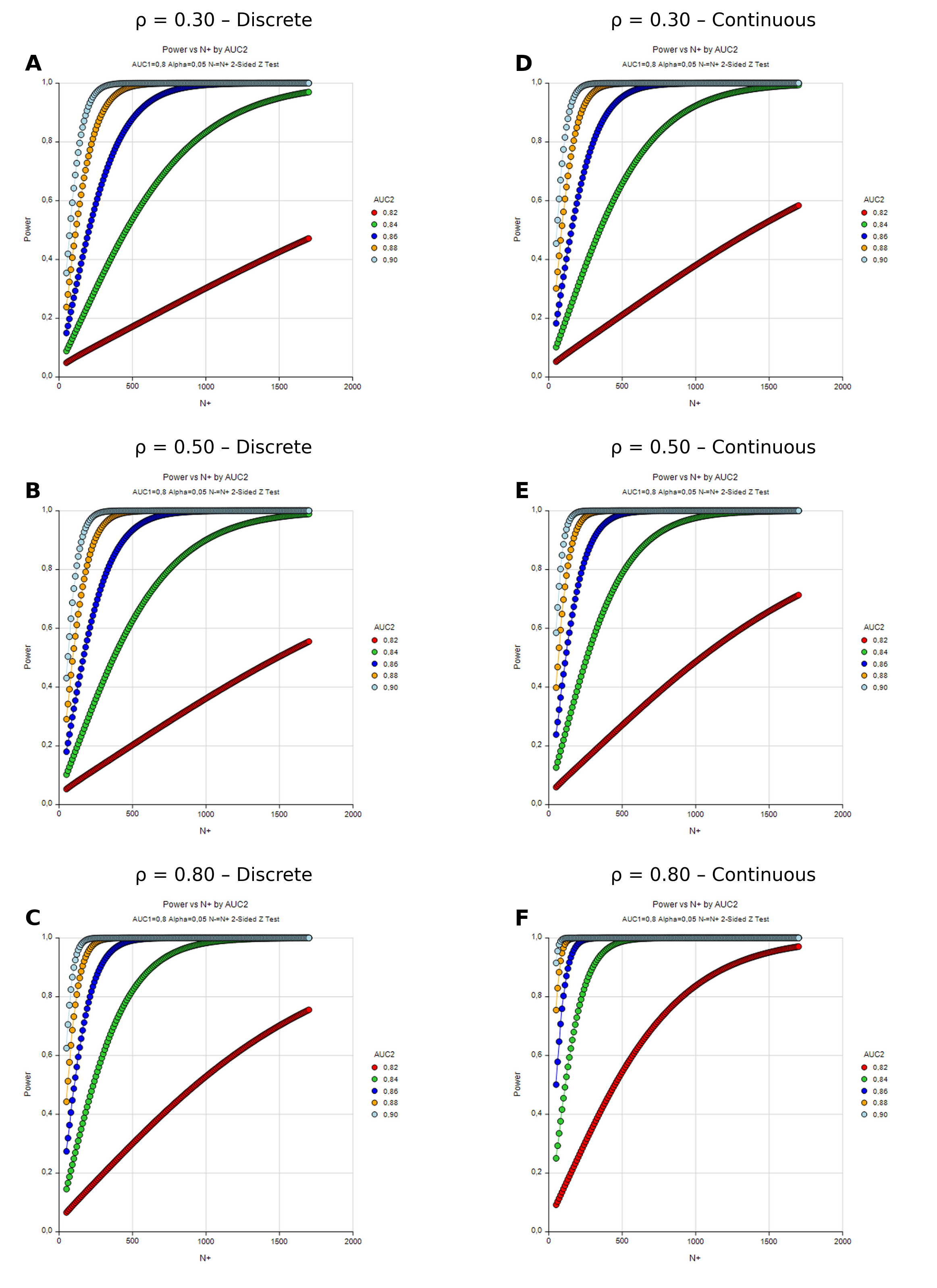

Supplement: Supplementary file 8 — Supplementary Material 8. [file 12874_2026_2768_MOESM8_ESM.jpeg]
