# Supplementary material for: Sample size and power analysis for ROC AUC differences in diagnostic tests: a methodological evaluation of the Obuchowski-McClish and Hanley-McNeil methods
Source: BMC Med Res Methodol. 2026 Jan 28;26:43. doi: 10.1186/s12874-026-02768-6 (PMC12924612; doi:10.1186/s12874-026-02768-6)
Supplement: Supplementary file 7 — Supplementary Material 7. [file 12874_2026_2768_MOESM7_ESM.docx]

**Supplementary Table S1** Sample size and statistical power for AUC comparisons (AUC₁=0.80 vs. AUC₂=0.86/0.88/0.90): Discrete data type, equal SD ratios (B_1_=B_2_=1.0), lower-upper FPR 0–1, positive, negative groups correlations 0.3.

| ΔAUC=0.06 | | | | | | | | | | ΔAUC=0.08 | | | | | | | | | | ΔAUC=0.10 | | | | | | | | |
| --- | --- | --- | --- | --- | --- | --- | --- | --- | --- | --- | --- | --- | --- | --- | --- | --- | --- | --- | --- | --- | --- | --- | --- | --- | --- | --- | --- | --- |
| Sample Size | | ED | | DISS | | |  | | | Sample Size | | | ED | | | DISS | |  | | Sample Size | | ED | | DISS | |  | |  |
| n^+^/n^-^ | n | D^+^/D^-^ | D | n^+'^/n^-'^ | n^'^ | TP | | AP | n^+^/n^-^ | | n | D^+^/D^-^ | | D | n^+'^/n^-'^ | | n^'^ | TP | AP | n^+^/n^-^ | n | D^+^/D^-^ | D | n^+'^/n^-'^ | n^'^ | | TP | AP |
| 307 | 614 | 77 | 154 | 384 | 768 | 0.680 | | 0.681 | 171 | | 342 | 43 | | 86 | 214 | | 428 | 0.680 | 0.680 | 109 | 218 | 28 | 56 | 137 | 274 | | 0.680 | 0.683 |
| 319 | 638 | 80 | 160 | 399 | 798 | 0.698 | | 0.698 | 178 | | 356 | 45 | | 90 | 223 | | 446 | 0.698 | 0.699 | 113 | 226 | 29 | 58 | 142 | 284 | | 0.698 | 0.700 |
| 332 | 664 | 83 | 166 | 415 | 830 | 0.715 | | 0.716 | 185 | | 370 | 47 | | 94 | 232 | | 464 | 0.715 | 0.716 | 117 | 234 | 30 | 60 | 147 | 294 | | 0.715 | 0.716 |
| 344 | 688 | 86 | 172 | 430 | 860 | 0.731 | | 0.732 | 192 | | 384 | 48 | | 96 | 240 | | 480 | 0.731 | 0.733 | 121 | 242 | 31 | 62 | 152 | 304 | | 0.731 | 0.732 |
| 356 | 712 | 89 | 178 | 445 | 890 | 0.746 | | 0.747 | 198 | | 396 | 50 | | 100 | 248 | | 496 | 0.746 | 0.747 | 125 | 250 | 32 | 64 | 157 | 314 | | 0.746 | 0.746 |
| 368 | 736 | 92 | 184 | 460 | 920 | 0.761 | | 0.761 | 205 | | 410 | 52 | | 104 | 257 | | 514 | 0.761 | 0.762 | 130 | 260 | 33 | 66 | 163 | 326 | | 0.761 | 0.764 |
| 380 | 760 | 95 | 190 | 475 | 950 | 0.774 | | 0.775 | 211 | | 422 | 53 | | 106 | 264 | | 528 | 0.774 | 0.775 | 134 | 268 | 34 | 68 | 168 | 336 | | 0.774 | 0.777 |
| 393 | 786 | 99 | 198 | 492 | 984 | 0.788 | | 0.789 | 218 | | 436 | 55 | | 110 | 273 | | 546 | 0.788 | 0.788 | 138 | 276 | 35 | 70 | 173 | 346 | | 0.788 | 0.790 |
| **404** | **808** | **101** | **202** | **505** | **1010** | **0.800** | | **0.800** | **225** | | **450** | **57** | | **114** | **282** | | **564** | **0.800** | **0.802** | **142** | **284** | **36** | **72** | **178** | **356** | | **0.800** | **0.802** |
| 416 | 832 | 104 | 208 | 520 | 1040 | 0.812 | | 0.812 | 231 | | 462 | 58 | | 116 | 289 | | 578 | 0.812 | 0.812 | 146 | 292 | 37 | 74 | 183 | 366 | | 0.812 | 0.813 |
| 428 | 856 | 107 | 214 | 535 | 1070 | 0.823 | | 0.823 | 238 | | 476 | 60 | | 120 | 298 | | 596 | 0.823 | 0.824 | 150 | 300 | 38 | 76 | 188 | 376 | | 0.823 | 0.824 |
| 441 | 882 | 111 | 222 | 552 | 1104 | 0.834 | | 0.835 | 245 | | 490 | 62 | | 124 | 307 | | 614 | 0.834 | 0.835 | 154 | 308 | 39 | 78 | 193 | 386 | | 0.834 | 0.835 |
| 465 | 930 | 117 | 234 | 582 | 1164 | 0.854 | | 0.854 | 258 | | 516 | 65 | | 130 | 323 | | 646 | 0.854 | 0.855 | 163 | 326 | 41 | 82 | 204 | 408 | | 0.854 | 0.856 |
| 478 | 956 | 120 | 240 | 598 | 1196 | 0.863 | | 0.864 | 265 | | 530 | 67 | | 134 | 332 | | 664 | 0.863 | 0.864 | 167 | 334 | 42 | 84 | 209 | 418 | | 0.863 | 0.865 |
| 489 | 978 | 123 | 246 | 612 | 1224 | 0.871 | | 0.871 | 271 | | 542 | 68 | | 136 | 339 | | 678 | 0.871 | 0.872 | 170 | 340 | 43 | 86 | 213 | 426 | | 0.871 | 0.871 |
| 501 | 1002 | 126 | 252 | 627 | 1254 | 0.879 | | 0.879 | 277 | | 554 | 70 | | 140 | 347 | | 694 | 0.879 | 0.879 | 174 | 348 | 44 | 88 | 218 | 436 | | 0.879 | 0.879 |
| 514 | 1028 | 129 | 258 | 643 | 1286 | 0.887 | | 0.888 | 284 | | 568 | 71 | | 142 | 355 | | 710 | 0.887 | 0.887 | 179 | 358 | 45 | 90 | 224 | 448 | | 0.887 | 0.888 |
| 525 | 1050 | 132 | 264 | 657 | 1314 | 0.894 | | 0.894 | 291 | | 582 | 73 | | 146 | 364 | | 728 | 0.894 | 0.895 | 183 | 366 | 46 | 92 | 229 | 458 | | 0.894 | 0.895 |
| **538** | **1076** | **135** | **270** | **673** | **1346** | **0.901** | | **0.901** | **298** | | **596** | **75** | | **150** | **373** | | **746** | **0.901** | **0.902** | **187** | **374** | **47** | **94** | **234** | **468** | | **0.901** | **0.902** |
| 549 | 1098 | 138 | 276 | 687 | 1374 | 0.907 | | 0.907 | 304 | | 608 | 76 | | 152 | 380 | | 760 | 0.907 | 0.908 | 191 | 382 | 48 | 96 | 239 | 478 | | 0.907 | 0.908 |
| 561 | 1122 | 141 | 282 | 702 | 1404 | 0.913 | | 0.913 | 310 | | 620 | 78 | | 156 | 388 | | 776 | 0.913 | 0.913 | 195 | 390 | 49 | 98 | 244 | 488 | | 0.913 | 0.914 |
| 574 | 1148 | 144 | 288 | 718 | 1436 | 0.919 | | 0.919 | 317 | | 634 | 80 | | 160 | 397 | | 794 | 0.919 | 0.919 | 199 | 398 | 50 | 100 | 249 | 498 | | 0.919 | 0.920 |
| 586 | 1172 | 147 | 294 | 733 | 1466 | 0.924 | | 0.924 | 324 | | 648 | 81 | | 162 | 405 | | 810 | 0.924 | 0.925 | 203 | 406 | 51 | 102 | 254 | 508 | | 0.924 | 0.925 |
| 598 | 1196 | 150 | 300 | 748 | 1496 | 0.929 | | 0.929 | 330 | | 660 | 83 | | 166 | 413 | | 826 | 0.929 | 0.929 | 207 | 414 | 52 | 104 | 259 | 518 | | 0.929 | 0.930 |
| 611 | 1222 | 153 | 306 | 764 | 1528 | 0.934 | | 0.934 | 337 | | 674 | 85 | | 170 | 422 | | 844 | 0.934 | 0.934 | 211 | 422 | 53 | 106 | 264 | 528 | | 0.934 | 0.934 |
| 622 | 1244 | 156 | 312 | 778 | 1556 | 0.938 | | 0.938 | 343 | | 686 | 86 | | 172 | 429 | | 858 | 0.938 | 0.938 | 215 | 430 | 54 | 108 | 269 | 538 | | 0.938 | 0.938 |
| 636 | 1272 | 159 | 318 | 795 | 1590 | 0.943 | | 0.943 | 351 | | 702 | 88 | | 176 | 439 | | 878 | 0.943 | 0.943 | 220 | 440 | 55 | 110 | 275 | 550 | | 0.943 | 0.944 |
| 646 | 1292 | 162 | 324 | 808 | 1616 | 0.946 | | 0.946 | 356 | | 712 | 89 | | 178 | 445 | | 890 | 0.946 | 0.946 | 223 | 446 | 56 | 112 | 279 | 558 | | 0.946 | 0.946 |
| **659** | **1318** | **165** | **330** | **824** | **1648** | **0.950** | | **0.950** | **364** | | **728** | **91** | | **182** | **455** | | **910** | **0.950** | **0.950** | **228** | **456** | **57** | **114** | **285** | **570** | | **0.950** | **0.951** |
| 670 | 1340 | 168 | 336 | 838 | 1676 | 0.953 | | 0.953 | 369 | | 738 | 93 | | 186 | 462 | | 924 | 0.953 | 0.953 | 231 | 462 | 58 | 116 | 289 | 578 | | 0.953 | 0.953 |
| 677 | 1354 | 170 | 340 | 847 | 1694 | 0.955 | | 0.955 | 373 | | 746 | 94 | | 188 | 467 | | 934 | 0.955 | 0.955 | 234 | 468 | 59 | 118 | 293 | 586 | | 0.955 | 0.956 |
| 697 | 1394 | 175 | 350 | 872 | 1744 | 0.960 | | 0.960 | 384 | | 768 | 96 | | 192 | 480 | | 960 | 0.960 | 0.960 | 240 | 480 | 60 | 120 | 300 | 600 | | 0.960 | 0.960 |
| 924 | 1848 | 231 | 462 | 1155 | 2310 | 0.990 | | 0.990 | 508 | | 1016 | 127 | | 254 | 635 | | 1270 | 0.990 | 0.990 | 316 | 632 | 79 | 158 | 395 | 790 | | 0.990 | 0.990 |
| **1273** | **2546** | **319** | **638** | **1592** | **3184** | **0.999** | | **0.999** | **698** | | **1396** | **175** | | **350** | **873** | | **1746** | **0.999** | **0.999** | **433** | **866** | **109** | **218** | **542** | **1084** | | **0.999** | **0.999** |

ED: expected number of dropouts, DISS: dropout-inflated enrollment sample size, TP: target power, AP: actual power

**Supplementary Table S2** Sample size and statistical power for AUC comparisons (AUC₁=0.80 vs. AUC₂=0.86/0.88/0.90): Continuous data type, lower-upper FPR 0–1, positive, negative groups correlations 0.3.

| ΔAUC=0.06 | | | | | | | | ΔAUC=0.08 | | | | | | | | ΔAUC=0.10 | | | | | | | | |
| --- | --- | --- | --- | --- | --- | --- | --- | --- | --- | --- | --- | --- | --- | --- | --- | --- | --- | --- | --- | --- | --- | --- | --- | --- |
| Sample Size | | ED | | DISS | |  |  | Sample Size | | ED | | DISS | |  |  | Sample Size | | ED | | DISS | |  |  |  |
| n^+^/n^-^ | n | D^+^/D^-^ | D | n^+'^/n^-'^ | n^'^ | TP | AP | n^+^/n^-^ | n | D^+^/D^-^ | D | n^+'^/n^-'^ | n^'^ | TP | AP | n^+^/n^-^ | n | D^+^/D^-^ | D | n^+'^/n^-'^ | n^'^ | TP | AP |  |
| 232 | 464 | 58 | 116 | 290 | 580 | 0.680 | 0.682 | 129 | 258 | 33 | 66 | 162 | 324 | 0.680 | 0.681 | 82 | 164 | 21 | 42 | 103 | 206 | 0.680 | 0.682 |  |
| 241 | 482 | 61 | 122 | 302 | 604 | 0.698 | 0.699 | 134 | 268 | 34 | 68 | 168 | 336 | 0.698 | 0.698 | 85 | 170 | 22 | 44 | 107 | 214 | 0.698 | 0.699 |  |
| 250 | 500 | 63 | 126 | 313 | 626 | 0.715 | 0.716 | 139 | 278 | 35 | 70 | 174 | 348 | 0.715 | 0.715 | 88 | 176 | 22 | 44 | 110 | 220 | 0.715 | 0.716 |  |
| 259 | 518 | 65 | 130 | 324 | 648 | 0.731 | 0.732 | 144 | 288 | 36 | 72 | 180 | 360 | 0.731 | 0.732 | 91 | 182 | 23 | 46 | 114 | 228 | 0.731 | 0.731 |  |
| 268 | 536 | 67 | 134 | 335 | 670 | 0.746 | 0.747 | 149 | 298 | 38 | 76 | 187 | 374 | 0.746 | 0.747 | 94 | 188 | 24 | 48 | 118 | 236 | 0.746 | 0.746 |  |
| 277 | 554 | 70 | 140 | 347 | 694 | 0.761 | 0.761 | 154 | 308 | 39 | 78 | 193 | 386 | 0.761 | 0.762 | 98 | 196 | 25 | 50 | 123 | 246 | 0.761 | 0.765 |  |
| 286 | 572 | 72 | 144 | 358 | 716 | 0.774 | 0.775 | 159 | 318 | 40 | 80 | 199 | 398 | 0.774 | 0.776 | 100 | 200 | 25 | 50 | 125 | 250 | 0.774 | 0.774 |  |
| 295 | 590 | 74 | 148 | 369 | 738 | 0.788 | 0.788 | 164 | 328 | 41 | 82 | 205 | 410 | 0.788 | 0.789 | 104 | 208 | 26 | 52 | 130 | 260 | 0.788 | 0.792 |  |
| **304** | **608** | **76** | **152** | **380** | **760** | **0.800** | **0.801** | **169** | **338** | **43** | **86** | **212** | **424** | **0.800** | **0.802** | **107** | **214** | **27** | **54** | **134** | **268** | **0.800** | **0.804** |  |
| 313 | 626 | 79 | 158 | 392 | 784 | 0.812 | 0.813 | 174 | 348 | 44 | 88 | 218 | 436 | 0.812 | 0.814 | 110 | 220 | 28 | 56 | 138 | 276 | 0.812 | 0.815 |  |
| 322 | 644 | 81 | 162 | 403 | 806 | 0.823 | 0.824 | 178 | 356 | 45 | 90 | 223 | 446 | 0.823 | 0.823 | 113 | 226 | 29 | 58 | 142 | 284 | 0.823 | 0.826 |  |
| 331 | 662 | 83 | 166 | 414 | 828 | 0.834 | 0.835 | 183 | 366 | 46 | 92 | 229 | 458 | 0.834 | 0.834 | 116 | 232 | 29 | 58 | 145 | 290 | 0.834 | 0.837 |  |
| 349 | 698 | 88 | 176 | 437 | 874 | 0.854 | 0.854 | 193 | 386 | 49 | 98 | 242 | 484 | 0.854 | 0.854 | 122 | 244 | 31 | 62 | 153 | 306 | 0.854 | 0.856 |  |
| 358 | 716 | 90 | 180 | 448 | 896 | 0.863 | 0.863 | 198 | 396 | 50 | 100 | 248 | 496 | 0.863 | 0.863 | 125 | 250 | 32 | 64 | 157 | 314 | 0.863 | 0.865 |  |
| 367 | 734 | 92 | 184 | 459 | 918 | 0.871 | 0.872 | 203 | 406 | 51 | 102 | 254 | 508 | 0.871 | 0.872 | 128 | 256 | 32 | 64 | 160 | 320 | 0.871 | 0.873 |  |
| 375 | 750 | 94 | 188 | 469 | 938 | 0.879 | 0.879 | 208 | 416 | 52 | 104 | 260 | 520 | 0.879 | 0.880 | 131 | 262 | 33 | 66 | 164 | 328 | 0.879 | 0.881 |  |
| 385 | 770 | 97 | 194 | 482 | 964 | 0.887 | 0.888 | 213 | 426 | 54 | 108 | 267 | 534 | 0.887 | 0.888 | 134 | 268 | 34 | 68 | 168 | 336 | 0.887 | 0.889 |  |
| 394 | 788 | 99 | 198 | 493 | 986 | 0.894 | 0.895 | 217 | 434 | 55 | 110 | 272 | 544 | 0.894 | 0.894 | 137 | 274 | 35 | 70 | 172 | 344 | 0.894 | 0.896 |  |
| **403** | **806** | **101** | **202** | **504** | **1008** | **0.901** | **0.901** | **222** | **444** | **56** | **112** | **278** | **556** | **0.901** | **0.901** | **140** | **280** | **35** | **70** | **175** | **350** | **0.901** | **0.903** |  |
| 411 | 822 | 103 | 206 | 514 | 1028 | 0.907 | 0.907 | 227 | 454 | 57 | 114 | 284 | 568 | 0.907 | 0.908 | 143 | 286 | 36 | 72 | 179 | 358 | 0.907 | 0.909 |  |
| 420 | 840 | 105 | 210 | 525 | 1050 | 0.913 | 0.913 | 232 | 464 | 58 | 116 | 290 | 580 | 0.913 | 0.914 | 146 | 292 | 37 | 74 | 183 | 366 | 0.913 | 0.915 |  |
| 430 | 860 | 108 | 216 | 538 | 1076 | 0.919 | 0.920 | 237 | 474 | 60 | 120 | 297 | 594 | 0.919 | 0.920 | 149 | 298 | 38 | 76 | 187 | 374 | 0.919 | 0.920 |  |
| 438 | 876 | 110 | 220 | 548 | 1096 | 0.924 | 0.924 | 242 | 484 | 61 | 122 | 303 | 606 | 0.924 | 0.925 | 152 | 304 | 38 | 76 | 190 | 380 | 0.924 | 0.926 |  |
| 447 | 894 | 112 | 224 | 559 | 1118 | 0.929 | 0.929 | 246 | 492 | 62 | 124 | 308 | 616 | 0.929 | 0.929 | 155 | 310 | 39 | 78 | 194 | 388 | 0.929 | 0.930 |  |
| 457 | 914 | 115 | 230 | 572 | 1144 | 0.934 | 0.934 | 252 | 504 | 63 | 126 | 315 | 630 | 0.934 | 0.935 | 158 | 316 | 40 | 80 | 198 | 396 | 0.934 | 0.935 |  |
| 465 | 930 | 117 | 234 | 582 | 1164 | 0.938 | 0.938 | 256 | 512 | 64 | 128 | 320 | 640 | 0.938 | 0.938 | 160 | 320 | 40 | 80 | 200 | 400 | 0.938 | 0.938 |  |
| 476 | 952 | 119 | 238 | 595 | 1190 | 0.943 | 0.943 | 262 | 524 | 66 | 132 | 328 | 656 | 0.943 | 0.943 | 164 | 328 | 41 | 82 | 205 | 410 | 0.943 | 0.944 |  |
| 482 | 964 | 121 | 242 | 603 | 1206 | 0.946 | 0.946 | 266 | 532 | 67 | 134 | 333 | 666 | 0.946 | 0.947 | 166 | 332 | 42 | 84 | 208 | 416 | 0.946 | 0.946 |  |
| **492** | **984** | **123** | **246** | **615** | **1230** | **0.950** | **0.950** | **271** | **542** | **68** | **136** | **339** | **678** | **0.950** | **0.950** | **170** | **340** | **43** | **86** | **213** | **426** | **0.950** | **0.951** |  |
| 500 | 1000 | 125 | 250 | 625 | 1250 | 0.953 | 0.953 | 275 | 550 | 69 | 138 | 344 | 688 | 0.953 | 0.953 | 172 | 344 | 43 | 86 | 215 | 430 | 0.953 | 0.953 |  |
| 506 | 1012 | 127 | 254 | 633 | 1266 | 0.955 | 0.955 | 278 | 556 | 70 | 140 | 348 | 696 | 0.955 | 0.955 | 174 | 348 | 44 | 88 | 218 | 436 | 0.955 | 0.955 |  |
| 520 | 1040 | 130 | 260 | 650 | 1300 | 0.960 | 0.960 | 286 | 572 | 72 | 144 | 358 | 716 | 0.960 | 0.960 | 179 | 358 | 45 | 90 | 224 | 448 | 0.960 | 0.960 |  |
| 688 | 1376 | 172 | 344 | 860 | 1720 | 0.990 | 0.990 | 377 | 754 | 95 | 190 | 472 | 944 | 0.990 | 0.990 | 235 | 470 | 59 | 118 | 294 | 588 | 0.990 | 0.990 |  |
| **945** | **1890** | **237** | **474** | **1182** | **2364** | **0.999** | **0.999** | **516** | **1032** | **129** | **258** | **645** | **1290** | **0.999** | **0.999** | **320** | **640** | **80** | **160** | **400** | **800** | **0.999** | **0.999** |  |

ED: expected number of dropouts, DISS: dropout-inflated enrollment sample size, TP: target power, AP: actual power

**Supplementary Table S3** Sample size and statistical power for AUC comparisons (AUC₁=0.80 vs. AUC₂=0.86/0.88/0.90): Discrete data type. equal SD ratios (B_1_=B_2_=1.0), lower-upper FPR 0–1, positive, negative groups correlations 0.5.

| ΔAUC=0.06 | | | | | | | | ΔAUC=0.08 | | | | | | | | ΔAUC=0.10 | | | | | | | |
| --- | --- | --- | --- | --- | --- | --- | --- | --- | --- | --- | --- | --- | --- | --- | --- | --- | --- | --- | --- | --- | --- | --- | --- |
| Sample Size | | ED | | DISS | |  |  | Sample Size | | ED | | DISS | |  |  | Sample Size | | ED | | DISS | |  |  |
| n^+^/n^-^ | n | D^+^/D^-^ | D | n^+'^/n^-'^ | n^'^ | TP | AP | n^+^/n^-^ | n | D^+^/D^-^ | D | n^+'^/n^-'^ | n^'^ | TP | AP | n^+^/n^-^ | n | D^+^/D^-^ | D | n^+'^/n^-'^ | n^'^ | TP | AP |
| 250 | 500 | 63 | 126 | 313 | 626 | 0.680 | 0.681 | 140 | 280 | 35 | 70 | 175 | 350 | 0.680 | 0.682 | 89 | 178 | 23 | 46 | 112 | 224 | 0.680 | 0.682 |
| 260 | 520 | 65 | 130 | 325 | 650 | 0.698 | 0.698 | 146 | 292 | 37 | 74 | 183 | 366 | 0.698 | 0.700 | 93 | 186 | 24 | 48 | 117 | 234 | 0.698 | 0.702 |
| 271 | 542 | 68 | 136 | 339 | 678 | 0.715 | 0.716 | 151 | 302 | 38 | 76 | 189 | 378 | 0.715 | 0.716 | 96 | 192 | 24 | 48 | 120 | 240 | 0.715 | 0.717 |
| 281 | 562 | 71 | 142 | 352 | 704 | 0.731 | 0.732 | 157 | 314 | 40 | 80 | 197 | 394 | 0.731 | 0.733 | 100 | 200 | 25 | 50 | 125 | 250 | 0.731 | 0.735 |
| 290 | 580 | 73 | 146 | 363 | 726 | 0.746 | 0.746 | 162 | 324 | 41 | 82 | 203 | 406 | 0.746 | 0.747 | 103 | 206 | 26 | 52 | 129 | 258 | 0.746 | 0.748 |
| 301 | 602 | 76 | 152 | 377 | 754 | 0.761 | 0.762 | 168 | 336 | 42 | 84 | 210 | 420 | 0.761 | 0.763 | 107 | 214 | 27 | 54 | 134 | 268 | 0.761 | 0.765 |
| 310 | 620 | 78 | 156 | 388 | 776 | 0.774 | 0.774 | 173 | 346 | 44 | 88 | 217 | 434 | 0.774 | 0.775 | 110 | 220 | 28 | 56 | 138 | 276 | 0.774 | 0.777 |
| 321 | 642 | 81 | 162 | 402 | 804 | 0.788 | 0.789 | 179 | 358 | 45 | 90 | 224 | 448 | 0.788 | 0.789 | 113 | 226 | 29 | 58 | 142 | 284 | 0.788 | 0.788 |
| **330** | **660** | **83** | **166** | **413** | **826** | **0.800** | **0.800** | **184** | **368** | **46** | **92** | **230** | **460** | **0.800** | **0.801** | **117** | **234** | **30** | **60** | **147** | **294** | **0.800** | **0.803** |
| 340 | 680 | 85 | 170 | 425 | 850 | 0.812 | 0.812 | 190 | 380 | 48 | 96 | 238 | 476 | 0.812 | 0.814 | 120 | 240 | 30 | 60 | 150 | 300 | 0.812 | 0.813 |
| 350 | 700 | 88 | 176 | 438 | 876 | 0.823 | 0.823 | 195 | 390 | 49 | 98 | 244 | 488 | 0.823 | 0.824 | 123 | 246 | 31 | 62 | 154 | 308 | 0.823 | 0.823 |
| 361 | 722 | 91 | 182 | 452 | 904 | 0.834 | 0.835 | 201 | 402 | 51 | 102 | 252 | 504 | 0.834 | 0.835 | 127 | 254 | 32 | 64 | 159 | 318 | 0.834 | 0.836 |
| 381 | 762 | 96 | 192 | 477 | 954 | 0.854 | 0.855 | 212 | 424 | 53 | 106 | 265 | 530 | 0.854 | 0.855 | 134 | 268 | 34 | 68 | 168 | 336 | 0.854 | 0.855 |
| 391 | 782 | 98 | 196 | 489 | 978 | 0.863 | 0.864 | 217 | 434 | 55 | 110 | 272 | 544 | 0.863 | 0.863 | 137 | 274 | 35 | 70 | 172 | 344 | 0.863 | 0.863 |
| 400 | 800 | 100 | 200 | 500 | 1000 | 0.871 | 0.871 | 222 | 444 | 56 | 112 | 278 | 556 | 0.871 | 0.871 | 141 | 282 | 36 | 72 | 177 | 354 | 0.871 | 0.873 |
| 410 | 820 | 103 | 206 | 513 | 1026 | 0.879 | 0.879 | 228 | 456 | 57 | 114 | 285 | 570 | 0.879 | 0.880 | 144 | 288 | 36 | 72 | 180 | 360 | 0.879 | 0.880 |
| 420 | 840 | 105 | 210 | 525 | 1050 | 0.887 | 0.887 | 234 | 468 | 59 | 118 | 293 | 586 | 0.887 | 0.888 | 148 | 296 | 37 | 74 | 185 | 370 | 0.887 | 0.889 |
| 430 | 860 | 108 | 216 | 538 | 1076 | 0.894 | 0.894 | 239 | 478 | 60 | 120 | 299 | 598 | 0.894 | 0.895 | 151 | 302 | 38 | 76 | 189 | 378 | 0.894 | 0.895 |
| **441** | **882** | **111** | **222** | **552** | **1104** | **0.901** | **0.902** | **245** | **490** | **62** | **124** | **307** | **614** | **0.901** | **0.902** | **154** | **308** | **39** | **78** | **193** | **386** | **0.901** | **0.901** |
| 450 | 900 | 113 | 226 | 563 | 1126 | 0.907 | 0.907 | 250 | 500 | 63 | 126 | 313 | 626 | 0.907 | 0.908 | 158 | 316 | 40 | 80 | 198 | 396 | 0.907 | 0.909 |
| 460 | 920 | 115 | 230 | 575 | 1150 | 0.913 | 0.913 | 255 | 510 | 64 | 128 | 319 | 638 | 0.913 | 0.913 | 161 | 322 | 41 | 82 | 202 | 404 | 0.913 | 0.914 |
| 470 | 940 | 118 | 236 | 588 | 1176 | 0.919 | 0.919 | 261 | 522 | 66 | 132 | 327 | 654 | 0.919 | 0.919 | 165 | 330 | 42 | 84 | 207 | 414 | 0.919 | 0.920 |
| 480 | 960 | 120 | 240 | 600 | 1200 | 0.924 | 0.924 | 266 | 532 | 67 | 134 | 333 | 666 | 0.924 | 0.924 | 168 | 336 | 42 | 84 | 210 | 420 | 0.924 | 0.925 |
| 490 | 980 | 123 | 246 | 613 | 1226 | 0.929 | 0.929 | 272 | 544 | 68 | 136 | 340 | 680 | 0.929 | 0.930 | 171 | 342 | 43 | 86 | 214 | 428 | 0.929 | 0.929 |
| 500 | 1000 | 125 | 250 | 625 | 1250 | 0.934 | 0.934 | 278 | 556 | 70 | 140 | 348 | 696 | 0.934 | 0.935 | 175 | 350 | 44 | 88 | 219 | 438 | 0.934 | 0.935 |
| 510 | 1020 | 128 | 256 | 638 | 1276 | 0.938 | 0.938 | 283 | 566 | 71 | 142 | 354 | 708 | 0.938 | 0.939 | 178 | 356 | 45 | 90 | 223 | 446 | 0.938 | 0.939 |
| 522 | 1044 | 131 | 262 | 653 | 1306 | 0.943 | 0.943 | 289 | 578 | 73 | 146 | 362 | 724 | 0.943 | 0.943 | 182 | 364 | 46 | 92 | 228 | 456 | 0.943 | 0.944 |
| 529 | 1058 | 133 | 266 | 662 | 1324 | 0.946 | 0.946 | 293 | 586 | 74 | 148 | 367 | 734 | 0.946 | 0.946 | 185 | 370 | 47 | 94 | 232 | 464 | 0.946 | 0.947 |
| **540** | **1080** | **135** | **270** | **675** | **1350** | **0.950** | **0.950** | **299** | **598** | **75** | **150** | **374** | **748** | **0.950** | **0.950** | **188** | **376** | **47** | **94** | **235** | **470** | **0.950** | **0.950** |
| 549 | 1098 | 138 | 276 | 687 | 1374 | 0.953 | 0.953 | 304 | 608 | 76 | 152 | 380 | 760 | 0.953 | 0.953 | 191 | 382 | 48 | 96 | 239 | 478 | 0.953 | 0.953 |
| 555 | 1110 | 139 | 278 | 694 | 1388 | 0.955 | 0.955 | 308 | 616 | 77 | 154 | 385 | 770 | 0.955 | 0.956 | 193 | 386 | 49 | 98 | 242 | 484 | 0.955 | 0.955 |
| 572 | 1144 | 143 | 286 | 715 | 1430 | 0.960 | 0.960 | 317 | 634 | 80 | 160 | 397 | 794 | 0.960 | 0.960 | 199 | 398 | 50 | 100 | 249 | 498 | 0.960 | 0.960 |
| 759 | 1518 | 190 | 380 | 949 | 1898 | 0.990 | 0.990 | 419 | 838 | 105 | 210 | 524 | 1048 | 0.990 | 0.990 | 263 | 526 | 66 | 132 | 329 | 658 | 0.990 | 0.990 |
| **1048** | **2096** | **262** | **524** | **1310** | **2620** | **0.999** | **0.999** | **577** | **1154** | **145** | **290** | **722** | **1444** | **0.999** | **0.999** | **361** | **722** | **91** | **182** | **452** | **904** | **0.999** | **0.999** |

ED: expected number of dropouts, DISS: dropout-inflated enrollment sample size, TP: target power, AP: actual power

**Supplementary Table S4** Sample size and statistical power for AUC comparisons (AUC₁=0.80 vs. AUC₂=0.86/0.88/0.90): Continuous data type, lower-upper FPR 0–1, positive, negative groups correlations 0.5.

| ΔAUC=0.06 | | | | | | | | ΔAUC=0.08 | | | | | | | | ΔAUC=0.10 | | | | | | | |
| --- | --- | --- | --- | --- | --- | --- | --- | --- | --- | --- | --- | --- | --- | --- | --- | --- | --- | --- | --- | --- | --- | --- | --- |
| Sample Size | | ED | | DISS | |  |  | Sample Size | | ED | | DISS | |  |  | Sample Size | | ED | | DISS | |  |  |
| n^+^/n^-^ | n | D^+^/D^-^ | D | n^+'^/n^-'^ | n^'^ | TP | AP | n^+^/n^-^ | n | D^+^/D^-^ | D | n^+'^/n^-'^ | n^'^ | TP | AP | n^+^/n^-^ | n | D^+^/D^-^ | D | n^+'^/n^-'^ | n^'^ | TP | AP |
| 173 | 346 | 44 | 88 | 217 | 434 | 0.680 | 0.681 | 97 | 194 | 25 | 50 | 122 | 244 | 0.680 | 0.683 | 62 | 124 | 16 | 32 | 78 | 156 | 0.680 | 0.686 |
| 180 | 360 | 45 | 90 | 225 | 450 | 0.698 | 0.699 | 101 | 202 | 26 | 52 | 127 | 254 | 0.698 | 0.702 | 64 | 128 | 16 | 32 | 80 | 160 | 0.698 | 0.701 |
| 187 | 374 | 47 | 94 | 234 | 468 | 0.715 | 0.717 | 104 | 208 | 26 | 52 | 130 | 260 | 0.715 | 0.716 | 66 | 132 | 17 | 34 | 83 | 166 | 0.715 | 0.716 |
| 194 | 388 | 49 | 98 | 243 | 486 | 0.731 | 0.733 | 108 | 216 | 27 | 54 | 135 | 270 | 0.731 | 0.733 | 69 | 138 | 18 | 36 | 87 | 174 | 0.731 | 0.736 |
| 200 | 400 | 50 | 100 | 250 | 500 | 0.746 | 0.747 | 112 | 224 | 28 | 56 | 140 | 280 | 0.746 | 0.749 | 71 | 142 | 18 | 36 | 89 | 178 | 0.746 | 0.749 |
| 207 | 414 | 52 | 104 | 259 | 518 | 0.761 | 0.762 | 116 | 232 | 29 | 58 | 145 | 290 | 0.761 | 0.765 | 73 | 146 | 19 | 38 | 92 | 184 | 0.761 | 0.762 |
| 214 | 428 | 54 | 108 | 268 | 536 | 0.774 | 0.776 | 119 | 238 | 30 | 60 | 149 | 298 | 0.774 | 0.776 | 76 | 152 | 19 | 38 | 95 | 190 | 0.774 | 0.780 |
| 221 | 442 | 56 | 112 | 277 | 554 | 0.788 | 0.789 | 123 | 246 | 31 | 62 | 154 | 308 | 0.788 | 0.790 | 78 | 156 | 20 | 40 | 98 | 196 | 0.788 | 0.791 |
| **227** | **454** | **57** | **114** | **284** | **568** | **0.800** | **0.801** | **126** | **252** | **32** | **64** | **158** | **316** | **0.800** | **0.800** | **80** | **160** | **20** | **40** | **100** | **200** | **0.800** | **0.802** |
| 234 | 468 | 59 | 118 | 293 | 586 | 0.812 | 0.813 | 130 | 260 | 33 | 66 | 163 | 326 | 0.812 | 0.813 | 83 | 166 | 21 | 42 | 104 | 208 | 0.812 | 0.817 |
| 241 | 482 | 61 | 122 | 302 | 604 | 0.823 | 0.824 | 134 | 268 | 34 | 68 | 168 | 336 | 0.823 | 0.825 | 85 | 170 | 22 | 44 | 107 | 214 | 0.823 | 0.827 |
| 248 | 496 | 62 | 124 | 310 | 620 | 0.834 | 0.835 | 138 | 276 | 35 | 70 | 173 | 346 | 0.834 | 0.837 | 87 | 174 | 22 | 44 | 109 | 218 | 0.834 | 0.836 |
| 261 | 522 | 66 | 132 | 327 | 654 | 0.854 | 0.854 | 145 | 290 | 37 | 74 | 182 | 364 | 0.854 | 0.855 | 92 | 184 | 23 | 46 | 115 | 230 | 0.854 | 0.857 |
| 268 | 536 | 67 | 134 | 335 | 670 | 0.863 | 0.864 | 149 | 298 | 38 | 76 | 187 | 374 | 0.863 | 0.865 | 94 | 188 | 24 | 48 | 118 | 236 | 0.863 | 0.865 |
| 274 | 548 | 69 | 138 | 343 | 686 | 0.871 | 0.871 | 152 | 304 | 38 | 76 | 190 | 380 | 0.871 | 0.872 | 96 | 192 | 24 | 48 | 120 | 240 | 0.871 | 0.872 |
| 281 | 562 | 71 | 142 | 352 | 704 | 0.879 | 0.880 | 156 | 312 | 39 | 78 | 195 | 390 | 0.879 | 0.880 | 98 | 196 | 25 | 50 | 123 | 246 | 0.879 | 0.879 |
| 288 | 576 | 72 | 144 | 360 | 720 | 0.887 | 0.888 | 160 | 320 | 40 | 80 | 200 | 400 | 0.887 | 0.889 | 101 | 202 | 26 | 52 | 127 | 254 | 0.887 | 0.889 |
| 295 | 590 | 74 | 148 | 369 | 738 | 0.894 | 0.895 | 163 | 326 | 41 | 82 | 204 | 408 | 0.894 | 0.894 | 103 | 206 | 26 | 52 | 129 | 258 | 0.894 | 0.895 |
| **301** | **602** | **76** | **152** | **377** | **754** | **0.901** | **0.901** | **167** | **334** | **42** | **84** | **209** | **418** | **0.901** | **0.902** | **105** | **210** | **27** | **54** | **132** | **264** | **0.901** | **0.901** |
| 308 | 616 | 77 | 154 | 385 | 770 | 0.907 | 0.908 | 171 | 342 | 43 | 86 | 214 | 428 | 0.907 | 0.909 | 108 | 216 | 27 | 54 | 135 | 270 | 0.907 | 0.909 |
| 314 | 628 | 79 | 158 | 393 | 786 | 0.913 | 0.913 | 174 | 348 | 44 | 88 | 218 | 436 | 0.913 | 0.914 | 110 | 220 | 28 | 56 | 138 | 276 | 0.913 | 0.915 |
| 322 | 644 | 81 | 162 | 403 | 806 | 0.919 | 0.920 | 178 | 356 | 45 | 90 | 223 | 446 | 0.919 | 0.920 | 112 | 224 | 28 | 56 | 140 | 280 | 0.919 | 0.919 |
| 328 | 656 | 82 | 164 | 410 | 820 | 0.924 | 0.924 | 182 | 364 | 46 | 92 | 228 | 456 | 0.924 | 0.925 | 114 | 228 | 29 | 58 | 143 | 286 | 0.924 | 0.924 |
| 335 | 670 | 84 | 168 | 419 | 838 | 0.929 | 0.930 | 185 | 370 | 47 | 94 | 232 | 464 | 0.929 | 0.929 | 117 | 234 | 30 | 60 | 147 | 294 | 0.929 | 0.931 |
| 342 | 684 | 86 | 172 | 428 | 856 | 0.934 | 0.934 | 189 | 378 | 48 | 96 | 237 | 474 | 0.934 | 0.935 | 119 | 238 | 30 | 60 | 149 | 298 | 0.934 | 0.935 |
| 348 | 696 | 87 | 174 | 435 | 870 | 0.938 | 0.938 | 192 | 384 | 48 | 96 | 240 | 480 | 0.938 | 0.938 | 121 | 242 | 31 | 62 | 152 | 304 | 0.938 | 0.939 |
| 356 | 712 | 89 | 178 | 445 | 890 | 0.943 | 0.943 | 197 | 394 | 50 | 100 | 247 | 494 | 0.943 | 0.944 | 124 | 248 | 31 | 62 | 155 | 310 | 0.943 | 0.944 |
| 361 | 722 | 91 | 182 | 452 | 904 | 0.946 | 0.946 | 200 | 400 | 50 | 100 | 250 | 500 | 0.946 | 0.947 | 126 | 252 | 32 | 64 | 158 | 316 | 0.946 | 0.947 |
| **369** | **738** | **93** | **186** | **462** | **924** | **0.950** | **0.950** | **204** | **408** | **51** | **102** | **255** | **510** | **0.950** | **0.951** | **128** | **256** | **32** | **64** | **160** | **320** | **0.950** | **0.950** |
| 375 | 750 | 94 | 188 | 469 | 938 | 0.953 | 0.953 | 207 | 414 | 52 | 104 | 259 | 518 | 0.953 | 0.954 | 130 | 260 | 33 | 66 | 163 | 326 | 0.953 | 0.953 |
| 379 | 758 | 95 | 190 | 474 | 948 | 0.955 | 0.955 | 209 | 418 | 53 | 106 | 262 | 524 | 0.955 | 0.955 | 132 | 264 | 33 | 66 | 165 | 330 | 0.955 | 0.956 |
| 390 | 780 | 98 | 196 | 488 | 976 | 0.960 | 0.960 | 215 | 430 | 54 | 108 | 269 | 538 | 0.960 | 0.960 | 135 | 270 | 34 | 68 | 169 | 338 | 0.960 | 0.960 |
| 515 | 1030 | 129 | 258 | 644 | 1288 | 0.990 | 0.990 | 284 | 568 | 71 | 142 | 355 | 710 | 0.990 | 0.990 | 178 | 356 | 45 | 90 | 223 | 446 | 0.990 | 0.990 |
| **709** | **1418** | **178** | **356** | **887** | **1774** | **0.999** | **0.999** | **389** | **778** | **98** | **196** | **487** | **974** | **0.999** | **0.999** | **243** | **486** | **61** | **122** | **304** | **608** | **0.999** | **0.999** |

ED: expected number of dropouts, DISS: dropout-inflated enrollment sample size, TP: target power, AP: actual power

**Supplementary Table S5** Sample size and statistical power for AUC comparisons (AUC₁=0.80 vs. AUC₂=0.86/0.88/0.90): Discrete data type. equal SD ratios (B_1_=B_2_=1.0), lower-upper FPR 0–1, positive, negative groups correlations 0.8.

| ΔAUC=0.06 | | | | | | | | ΔAUC=0.08 | | | | | | | | ΔAUC=0.10 | | | | | | | |
| --- | --- | --- | --- | --- | --- | --- | --- | --- | --- | --- | --- | --- | --- | --- | --- | --- | --- | --- | --- | --- | --- | --- | --- |
| Sample Size | | ED | | DISS | |  |  | Sample Size | | ED | | DISS | |  |  | Sample Size | | ED | | DISS | |  |  |
| n^+^/n^-^ | n | D^+^/D^-^ | D | n^+'^/n^-'^ | n^'^ | TP | AP | n^+^/n^-^ | n | D^+^/D^-^ | D | n^+'^/n^-'^ | n^'^ | TP | AP | n^+^/n^-^ | n | D^+^/D^-^ | D | n^+'^/n^-'^ | n^'^ | TP | AP |
| 159 | 318 | 40 | 80 | 199 | 398 | 0.680 | 0.683 | 89 | 178 | 23 | 46 | 112 | 224 | 0.680 | 0.681 | 57 | 114 | 15 | 30 | 72 | 144 | 0.680 | 0.683 |
| 165 | 330 | 42 | 84 | 207 | 414 | 0.698 | 0.699 | 93 | 186 | 24 | 48 | 117 | 234 | 0.698 | 0.701 | 60 | 120 | 15 | 30 | 75 | 150 | 0.698 | 0.705 |
| 172 | 344 | 43 | 86 | 215 | 430 | 0.715 | 0.717 | 97 | 194 | 25 | 50 | 122 | 244 | 0.715 | 0.719 | 62 | 124 | 16 | 32 | 78 | 156 | 0.715 | 0.719 |
| 178 | 356 | 45 | 90 | 223 | 446 | 0.731 | 0.732 | 100 | 200 | 25 | 50 | 125 | 250 | 0.731 | 0.732 | 64 | 128 | 16 | 32 | 80 | 160 | 0.731 | 0.733 |
| 185 | 370 | 47 | 94 | 232 | 464 | 0.746 | 0.748 | 104 | 208 | 26 | 52 | 130 | 260 | 0.746 | 0.749 | 66 | 132 | 17 | 34 | 83 | 166 | 0.746 | 0.746 |
| 191 | 382 | 48 | 96 | 239 | 478 | 0.761 | 0.761 | 108 | 216 | 27 | 54 | 135 | 270 | 0.761 | 0.765 | 69 | 138 | 18 | 36 | 87 | 174 | 0.761 | 0.765 |
| 197 | 394 | 50 | 100 | 247 | 494 | 0.774 | 0.774 | 111 | 222 | 28 | 56 | 139 | 278 | 0.774 | 0.776 | 71 | 142 | 18 | 36 | 89 | 178 | 0.774 | 0.777 |
| 204 | 408 | 51 | 102 | 255 | 510 | 0.788 | 0.788 | 115 | 230 | 29 | 58 | 144 | 288 | 0.788 | 0.790 | 73 | 146 | 19 | 38 | 92 | 184 | 0.788 | 0.788 |
| **211** | **422** | **53** | **106** | **264** | **528** | **0.800** | **0.802** | **118** | **236** | **30** | **60** | **148** | **296** | **0.800** | **0.801** | **76** | **152** | **19** | **38** | **95** | **190** | **0.800** | **0.804** |
| 217 | 434 | 55 | 110 | 272 | 544 | 0.812 | 0.813 | 122 | 244 | 31 | 62 | 153 | 306 | 0.812 | 0.814 | 78 | 156 | 20 | 40 | 98 | 196 | 0.812 | 0.815 |
| 223 | 446 | 56 | 112 | 279 | 558 | 0.823 | 0.823 | 125 | 250 | 32 | 64 | 157 | 314 | 0.823 | 0.823 | 80 | 160 | 20 | 40 | 100 | 200 | 0.823 | 0.824 |
| 230 | 460 | 58 | 116 | 288 | 576 | 0.834 | 0.835 | 129 | 258 | 33 | 66 | 162 | 324 | 0.834 | 0.835 | 83 | 166 | 21 | 42 | 104 | 208 | 0.834 | 0.838 |
| 243 | 486 | 61 | 122 | 304 | 608 | 0.854 | 0.854 | 137 | 274 | 35 | 70 | 172 | 344 | 0.854 | 0.856 | 87 | 174 | 22 | 44 | 109 | 218 | 0.854 | 0.855 |
| 250 | 500 | 63 | 126 | 313 | 626 | 0.863 | 0.864 | 140 | 280 | 35 | 70 | 175 | 350 | 0.863 | 0.864 | 90 | 180 | 23 | 46 | 113 | 226 | 0.863 | 0.866 |
| 256 | 512 | 64 | 128 | 320 | 640 | 0.871 | 0.872 | 144 | 288 | 36 | 72 | 180 | 360 | 0.871 | 0.873 | 92 | 184 | 23 | 46 | 115 | 230 | 0.871 | 0.874 |
| 262 | 524 | 66 | 132 | 328 | 656 | 0.879 | 0.879 | 147 | 294 | 37 | 74 | 184 | 368 | 0.879 | 0.880 | 94 | 188 | 24 | 48 | 118 | 236 | 0.879 | 0.881 |
| 269 | 538 | 68 | 136 | 337 | 674 | 0.887 | 0.887 | 151 | 302 | 38 | 76 | 189 | 378 | 0.887 | 0.888 | 96 | 192 | 24 | 48 | 120 | 240 | 0.887 | 0.887 |
| 275 | 550 | 69 | 138 | 344 | 688 | 0.894 | 0.894 | 155 | 310 | 39 | 78 | 194 | 388 | 0.894 | 0.896 | 99 | 198 | 25 | 50 | 124 | 248 | 0.894 | 0.896 |
| **282** | **564** | **71** | **142** | **353** | **706** | **0.901** | **0.901** | **158** | **316** | **40** | **80** | **198** | **396** | **0.901** | **0.901** | **101** | **202** | **26** | **52** | **127** | **254** | **0.901** | **0.902** |
| 288 | 576 | 72 | 144 | 360 | 720 | 0.907 | 0.907 | 162 | 324 | 41 | 82 | 203 | 406 | 0.907 | 0.908 | 103 | 206 | 26 | 52 | 129 | 258 | 0.907 | 0.908 |
| 295 | 590 | 74 | 148 | 369 | 738 | 0.913 | 0.914 | 165 | 330 | 42 | 84 | 207 | 414 | 0.913 | 0.913 | 106 | 212 | 27 | 54 | 133 | 266 | 0.913 | 0.915 |
| 302 | 604 | 76 | 152 | 378 | 756 | 0.919 | 0.920 | 169 | 338 | 43 | 86 | 212 | 424 | 0.919 | 0.919 | 108 | 216 | 27 | 54 | 135 | 270 | 0.919 | 0.920 |
| 308 | 616 | 77 | 154 | 385 | 770 | 0.924 | 0.925 | 173 | 346 | 44 | 88 | 217 | 434 | 0.924 | 0.925 | 110 | 220 | 28 | 56 | 138 | 276 | 0.924 | 0.925 |
| 314 | 628 | 79 | 158 | 393 | 786 | 0.929 | 0.929 | 176 | 352 | 44 | 88 | 220 | 440 | 0.929 | 0.929 | 112 | 224 | 28 | 56 | 140 | 280 | 0.929 | 0.929 |
| 321 | 642 | 81 | 162 | 402 | 804 | 0.934 | 0.934 | 180 | 360 | 45 | 90 | 225 | 450 | 0.934 | 0.934 | 115 | 230 | 29 | 58 | 144 | 288 | 0.934 | 0.935 |
| 327 | 654 | 82 | 164 | 409 | 818 | 0.938 | 0.938 | 183 | 366 | 46 | 92 | 229 | 458 | 0.938 | 0.938 | 117 | 234 | 30 | 60 | 147 | 294 | 0.938 | 0.939 |
| 335 | 670 | 84 | 168 | 419 | 838 | 0.943 | 0.943 | 188 | 376 | 47 | 94 | 235 | 470 | 0.943 | 0.944 | 120 | 240 | 30 | 60 | 150 | 300 | 0.943 | 0.944 |
| 340 | 680 | 85 | 170 | 425 | 850 | 0.946 | 0.946 | 191 | 382 | 48 | 96 | 239 | 478 | 0.946 | 0.947 | 122 | 244 | 31 | 62 | 153 | 306 | 0.946 | 0.947 |
| **347** | **694** | **87** | **174** | **434** | **868** | **0.950** | **0.950** | **195** | **390** | **49** | **98** | **244** | **488** | **0.950** | **0.951** | **124** | **248** | **31** | **62** | **155** | **310** | **0.950** | **0.950** |
| 353 | 706 | 89 | 178 | 442 | 884 | 0.953 | 0.953 | 198 | 396 | 50 | 100 | 248 | 496 | 0.953 | 0.954 | 126 | 252 | 32 | 64 | 158 | 316 | 0.953 | 0.953 |
| 357 | 714 | 90 | 180 | 447 | 894 | 0.955 | 0.955 | 200 | 400 | 50 | 100 | 250 | 500 | 0.955 | 0.955 | 128 | 256 | 32 | 64 | 160 | 320 | 0.955 | 0.956 |
| 368 | 736 | 92 | 184 | 460 | 920 | 0.960 | 0.960 | 206 | 412 | 52 | 104 | 258 | 516 | 0.960 | 0.960 | 131 | 262 | 33 | 66 | 164 | 328 | 0.960 | 0.960 |
| 490 | 980 | 123 | 246 | 613 | 1226 | 0.990 | 0.990 | 274 | 548 | 69 | 138 | 343 | 686 | 0.990 | 0.990 | 175 | 350 | 44 | 88 | 219 | 438 | 0.990 | 0.990 |
| **679** | **1358** | **170** | **340** | **849** | **1698** | **0.999** | **0.999** | **380** | **760** | **95** | **190** | **475** | **950** | **0.999** | **0.999** | **242** | **484** | **61** | **122** | **303** | **606** | **0.999** | **0.999** |

ED: expected number of dropouts, DISS: dropout-inflated enrollment sample size, TP: target power, AP: actual power

**Supplementary Table S6** Sample size and statistical power for AUC comparisons (AUC₁=0.80 vs. AUC₂=0.86/0.88/0.90): Continuous data type, lower-upper FPR 0–1, positive, negative groups correlations 0.8.

| ΔAUC=0.06 | | | | | | | | ΔAUC=0.08 | | | | | | | | ΔAUC=0.10 | | | | | | | |
| --- | --- | --- | --- | --- | --- | --- | --- | --- | --- | --- | --- | --- | --- | --- | --- | --- | --- | --- | --- | --- | --- | --- | --- |
| Sample Size | | ED | | DISS | |  |  | Sample Size | | ED | | DISS | |  |  | Sample Size | | ED | | DISS | |  |  |
| n^+^/n^-^ | n | D^+^/D^-^ | D | n^+'^/n^-'^ | n^'^ | TP | AP | n^+^/n^-^ | n | D^+^/D^-^ | D | n^+'^/n^-'^ | n^'^ | TP | AP | n^+^/n^-^ | n | D^+^/D^-^ | D | n^+'^/n^-'^ | n^'^ | TP | AP |
| 76 | 152 | 19 | 38 | 95 | 190 | 0.680 | 0.684 | 43 | 86 | 11 | 22 | 54 | 108 | 0.680 | 0.688 | 28 | 56 | 7 | 14 | 35 | 70 | 0.680 | 0.695 |
| 79 | 158 | 20 | 40 | 99 | 198 | 0.698 | 0.702 | 45 | 90 | 12 | 24 | 57 | 114 | 0.698 | 0.708 | 29 | 58 | 8 | 16 | 37 | 74 | 0.698 | 0.710 |
| 82 | 164 | 21 | 42 | 103 | 206 | 0.715 | 0.718 | 46 | 92 | 12 | 24 | 58 | 116 | 0.715 | 0.718 | 30 | 60 | 8 | 16 | 38 | 76 | 0.715 | 0.726 |
| 85 | 170 | 22 | 44 | 107 | 214 | 0.731 | 0.734 | 48 | 96 | 12 | 24 | 60 | 120 | 0.731 | 0.737 | 31 | 62 | 8 | 16 | 39 | 78 | 0.731 | 0.740 |
| 88 | 176 | 22 | 44 | 110 | 220 | 0.746 | 0.749 | 50 | 100 | 13 | 26 | 63 | 126 | 0.746 | 0.754 | 32 | 64 | 8 | 16 | 40 | 80 | 0.746 | 0.754 |
| 91 | 182 | 23 | 46 | 114 | 228 | 0.761 | 0.764 | 51 | 102 | 13 | 26 | 64 | 128 | 0.761 | 0.763 | 33 | 66 | 9 | 18 | 42 | 84 | 0.761 | 0.767 |
| 94 | 188 | 24 | 48 | 118 | 236 | 0.774 | 0.777 | 53 | 106 | 14 | 28 | 67 | 134 | 0.774 | 0.779 | 34 | 68 | 9 | 18 | 43 | 86 | 0.774 | 0.779 |
| 97 | 194 | 25 | 50 | 122 | 244 | 0.788 | 0.790 | 55 | 110 | 14 | 28 | 69 | 138 | 0.788 | 0.795 | 35 | 70 | 9 | 18 | 44 | 88 | 0.788 | 0.791 |
| **100** | **200** | **25** | **50** | **125** | **250** | **0.800** | **0.803** | **56** | **112** | **14** | **28** | **70** | **140** | **0.800** | **0.802** | **36** | **72** | **9** | **18** | **45** | **90** | **0.800** | **0.803** |
| 103 | 206 | 26 | 52 | 129 | 258 | 0.812 | 0.814 | 58 | 116 | 15 | 30 | 73 | 146 | 0.812 | 0.816 | 37 | 74 | 10 | 20 | 47 | 94 | 0.812 | 0.814 |
| 106 | 212 | 27 | 54 | 133 | 266 | 0.823 | 0.826 | 60 | 120 | 15 | 30 | 75 | 150 | 0.823 | 0.829 | 38 | 76 | 10 | 20 | 48 | 96 | 0.823 | 0.824 |
| 109 | 218 | 28 | 56 | 137 | 274 | 0.834 | 0.836 | 61 | 122 | 16 | 32 | 77 | 154 | 0.834 | 0.835 | 39 | 78 | 10 | 20 | 49 | 98 | 0.834 | 0.834 |
| 115 | 230 | 29 | 58 | 144 | 288 | 0.854 | 0.856 | 65 | 130 | 17 | 34 | 82 | 164 | 0.854 | 0.859 | 42 | 84 | 11 | 22 | 53 | 106 | 0.854 | 0.861 |
| 118 | 236 | 30 | 60 | 148 | 296 | 0.863 | 0.865 | 66 | 132 | 17 | 34 | 83 | 166 | 0.863 | 0.864 | 43 | 86 | 11 | 22 | 54 | 108 | 0.863 | 0.869 |
| 121 | 242 | 31 | 62 | 152 | 304 | 0.871 | 0.873 | 68 | 136 | 17 | 34 | 85 | 170 | 0.871 | 0.874 | 44 | 88 | 11 | 22 | 55 | 110 | 0.871 | 0.877 |
| 124 | 248 | 31 | 62 | 155 | 310 | 0.879 | 0.881 | 70 | 140 | 18 | 36 | 88 | 176 | 0.879 | 0.883 | 45 | 90 | 12 | 24 | 57 | 114 | 0.879 | 0.884 |
| 127 | 254 | 32 | 64 | 159 | 318 | 0.887 | 0.889 | 71 | 142 | 18 | 36 | 89 | 178 | 0.887 | 0.888 | 46 | 92 | 12 | 24 | 58 | 116 | 0.887 | 0.891 |
| 130 | 260 | 33 | 66 | 163 | 326 | 0.894 | 0.896 | 73 | 146 | 19 | 38 | 92 | 184 | 0.894 | 0.896 | 47 | 94 | 12 | 24 | 59 | 118 | 0.894 | 0.897 |
| **133** | **266** | **34** | **68** | **167** | **334** | **0.901** | **0.902** | **75** | **150** | **19** | **38** | **94** | **188** | **0.901** | **0.904** | **48** | **96** | **12** | **24** | **60** | **120** | **0.901** | **0.903** |
| 136 | 272 | 34 | 68 | 170 | 340 | 0.907 | 0.909 | 76 | 152 | 19 | 38 | 95 | 190 | 0.907 | 0.908 | 49 | 98 | 13 | 26 | 62 | 124 | 0.907 | 0.909 |
| 139 | 278 | 35 | 70 | 174 | 348 | 0.913 | 0.915 | 78 | 156 | 20 | 40 | 98 | 196 | 0.913 | 0.915 | 50 | 100 | 13 | 26 | 63 | 126 | 0.913 | 0.915 |
| 142 | 284 | 36 | 72 | 178 | 356 | 0.919 | 0.920 | 80 | 160 | 20 | 40 | 100 | 200 | 0.919 | 0.922 | 51 | 102 | 13 | 26 | 64 | 128 | 0.919 | 0.920 |
| 145 | 290 | 37 | 74 | 182 | 364 | 0.924 | 0.926 | 81 | 162 | 21 | 42 | 102 | 204 | 0.924 | 0.925 | 52 | 104 | 13 | 26 | 65 | 130 | 0.924 | 0.925 |
| 148 | 296 | 37 | 74 | 185 | 370 | 0.929 | 0.930 | 83 | 166 | 21 | 42 | 104 | 208 | 0.929 | 0.931 | 53 | 106 | 14 | 28 | 67 | 134 | 0.929 | 0.929 |
| 151 | 302 | 38 | 76 | 189 | 378 | 0.934 | 0.935 | 85 | 170 | 22 | 44 | 107 | 214 | 0.934 | 0.936 | 55 | 110 | 14 | 28 | 69 | 138 | 0.934 | 0.938 |
| 154 | 308 | 39 | 78 | 193 | 386 | 0.938 | 0.939 | 86 | 172 | 22 | 44 | 108 | 216 | 0.938 | 0.939 | 56 | 112 | 14 | 28 | 70 | 140 | 0.938 | 0.942 |
| 157 | 314 | 40 | 80 | 197 | 394 | 0.943 | 0.943 | 88 | 176 | 22 | 44 | 110 | 220 | 0.943 | 0.944 | 57 | 114 | 15 | 30 | 72 | 144 | 0.943 | 0.945 |
| 159 | 318 | 40 | 80 | 199 | 398 | 0.946 | 0.946 | 90 | 180 | 23 | 46 | 113 | 226 | 0.946 | 0.948 | 58 | 116 | 15 | 30 | 73 | 146 | 0.946 | 0.949 |
| **163** | **326** | **41** | **82** | **204** | **408** | **0.950** | **0.951** | **91** | **182** | **23** | **46** | **114** | **228** | **0.950** | **0.950** | **59** | **118** | **15** | **30** | **74** | **148** | **0.950** | **0.952** |
| 165 | 330 | 42 | 84 | 207 | 414 | 0.953 | 0.953 | 93 | 186 | 24 | 48 | 117 | 234 | 0.953 | 0.954 | 60 | 120 | 15 | 30 | 75 | 150 | 0.953 | 0.955 |
| 167 | 334 | 42 | 84 | 209 | 418 | 0.955 | 0.955 | 94 | 188 | 24 | 48 | 118 | 236 | 0.955 | 0.956 | 60 | 120 | 15 | 30 | 75 | 150 | 0.955 | 0.955 |
| 172 | 344 | 43 | 86 | 215 | 430 | 0.960 | 0.960 | 97 | 194 | 25 | 50 | 122 | 244 | 0.960 | 0.961 | 62 | 124 | 16 | 32 | 78 | 156 | 0.960 | 0.961 |
| 228 | 456 | 57 | 114 | 285 | 570 | 0.990 | 0.990 | 128 | 256 | 32 | 64 | 160 | 320 | 0.990 | 0.990 | 82 | 164 | 21 | 42 | 103 | 206 | 0.990 | 0.990 |
| **315** | **630** | **79** | **158** | **394** | **788** | **0.999** | **0.999** | **176** | **352** | **44** | **88** | **220** | **440** | **0.999** | **0.999** | **113** | **226** | **29** | **58** | **142** | **284** | **0.999** | **0.999** |

ED: expected number of dropouts, DISS: dropout-inflated enrollment sample size, TP: target power, AP: actual power
